# The sympathetic nervous system exacerbates carotid body sensitivity in hypertension

**DOI:** 10.1093/cvr/cvac008

**Published:** 2022-01-20

**Authors:** Igor S A Felippe, Tymoteusz Zera, Melina P da Silva, Davi J A Moraes, Fiona McBryde, Julian F R Paton

**Affiliations:** Department of Physiology, Faculty of Health & Medical Sciences, Manaaki Mānawa—The Centre for Heart Research, University of Auckland , 85 Park Road, Grafton Campus, Auckland 1023, New Zealand; Department of Experimental and Clinical Physiology, Laboratory of Centre for Preclinical Research, Medical University of Warsaw, Warsaw 02-091, Poland; Department of Physiology, School of Medicine of Ribeirão Preto, University of São Paulo, Ribeirão Preto, SP 14040-900, Brazil; Department of Physiology, School of Medicine of Ribeirão Preto, University of São Paulo, Ribeirão Preto, SP 14040-900, Brazil; Department of Physiology, Faculty of Health & Medical Sciences, Manaaki Mānawa—The Centre for Heart Research, University of Auckland , 85 Park Road, Grafton Campus, Auckland 1023, New Zealand; Department of Physiology, Faculty of Health & Medical Sciences, Manaaki Mānawa—The Centre for Heart Research, University of Auckland , 85 Park Road, Grafton Campus, Auckland 1023, New Zealand

**Keywords:** Carotid body, Superior cervical ganglion, Chemoreceptors, Autonomic nervous system, α_1_-Adrenoreceptors

## Abstract

**Aims:**

The carotid bodies (CBs) of spontaneously hypertensive (SH) rats exhibit hypertonicity and hyperreflexia contributing to heightened peripheral sympathetic outflow. We hypothesized that CB hyperexcitability is driven by its own sympathetic innervation.

**Methods and results:**

To test this, the chemoreflex was activated (NaCN 50–100 µL, 0.4 µg/µL) in SH and Wistar rats *in situ* before and after: (i) electrical stimulation (ES; 30 Hz, 2 ms, 10 V) of the superior cervical ganglion (SCG), which innervates the CB; (ii) unilateral resection of the SCG (SCGx); (iii) CB injections of an α_1_-adrenergic receptor agonist (phenylephrine, 50 µL, 1 mmol/L), and (iv) α_1_-adrenergic receptor antagonist prazosin (40 µL, 1 mmol/L) or tamsulosin (50 µL, 1 mmol/L). ES of the SCG enhanced CB-evoked sympathoexcitation by 40–50% (*P* < 0.05) with no difference between rat strains. Unilateral SCGx attenuated the CB-evoked sympathoexcitation in SH (62%; *P* < 0.01) but was without effect in Wistar rats; it also abolished the ongoing firing of chemoreceptive petrosal neurones of SH rats, which became hyperpolarized. In Wistar rats, CB injections of phenylephrine enhanced CB-evoked sympathoexcitation (33%; *P* < 0.05), which was prevented by prazosin (26%; *P* < 0.05) in SH rats. Tamsulosin alone reproduced the effects of prazosin in SH rats and prevented the sensitizing effect of the SCG following ES. Within the CB, α_1A_- and α_1B_-adrenoreceptors were co-localized on both glomus cells and blood vessels. In conscious SH rats instrumented for recording blood pressure (BP), the CB-evoked pressor response was attenuated after SCGx, and systolic BP fell by 16 ± 4.85 mmHg.

**Conclusions:**

The sympathetic innervation of the CB is tonically activated and sensitizes the CB of SH but not Wistar rats. Furthermore, sensitization of CB-evoked reflex sympathoexcitation appears to be mediated by α_1_-adrenoceptors located either on the vasculature and/or glomus cells. The SCG is novel target for controlling CB pathophysiology in hypertension.

## Graphical Abstract

**Graphical Abstract:**
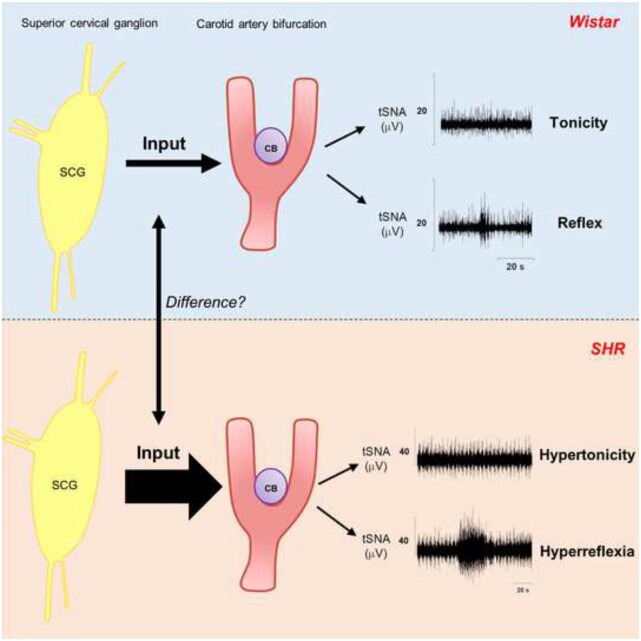

## 1. Introduction

Hypertension is a major global problem that affects more than 1 billion people worldwide.^1^ High blood pressure (BP) is the single most important risk factor for cardiovascular death globally due to cardiovascular diseases such as haemorrhagic and ischaemic stroke, ischaemic heart disease, and heart failure.^2,3^ Arterial hypertension has been independently associated with severe coronavirus disease (COVID-19) and mortality in patients infected with severe acute respiratory syndrome coronavirus 2 (SARS-CoV-2).^4,5^ Thus, unveiling the mechanisms underlying the development and maintenance of hypertension remains pivotal.

The carotid body (CB) is the major peripheral chemoreceptor organ, sensing blood oxygenation^6^ but also other modalities.^7–10^ Our group has demonstrated that the CB is involved with both the development and maintenance of neurogenic hypertension that is associated with pathological development of both hyperreflexia and hypertonicity, so-called CB hyperexcitability.^11–13^ CB denervation or its resection has been shown to be an effective way of treating hypertension in animal models^11,13,14^ and a subset of human patients.^15^ Understanding the mechanisms that drive CB hyperexcitability has now become crucial to inform prospective targets for new drugs. Pijacka *et al*.^13^ described the upregulation and functional importance of purinergic P2X3 receptors in driving CB hyperexcitability in hypertension whereas other mechanisms exist in different disease states.^16–18^

In heart failure, reduced CB blood flow has been proposed as a mechanism that heightens CB sensitivity.^19^ This is supported by studies showing that manipulation of CB vascular tone via its autonomic innervation (parasympathetic-vasodilatation^20^ and sympathetic-vasoconstriction^21^) is associated with concordant changes in CB afferent discharge.^22^ Floyd and Neil^23^ demonstrated that stimulating the sympathetic efferent nerves to the CB increased CB afferent discharge, although it remains unknown whether this resulted in changes in the magnitude of evoked reflex responses. O’Regan^24^ proposed that stimulating the sympathetic efferents to the CB increases chemoreceptor discharge by both vascular and non-vascular mechanisms including: (i) vascular α_1_-adrenoreceptors causing vasoconstriction^21^ and (ii) a non-vascular effect^25^ that enhances release of neurotransmitters (e.g. ATP) from glomus cells.^22,25^ However, whether any of these mechanisms play a role in driving chronic CB excitability in disease states is unknown.

In hypertension, we hypothesize that CB hyperexcitability is driven by excessive activity of its sympathetic innervation. Since the sympathetic innervation of the CB originates primarily from the superior cervical ganglion (SCG^26^), we assessed whether either resecting the SCG or antagonizing α_1_-adrenoreceptors within the CB would reduce the augmented chemoreflex-evoked responses in spontaneously hypertensive (SH) rats. Our results present the first evidence for a causal role of the SCG in mediating chemoreflex hypertonicity and enhanced evoked motor responses in a hypertensive animal model.

## 2. Methods

### 2.1 Animals

Male Wistar and SH rats (*Rattus norvegicus*) were bred by the Vernon Jansen unit of the University of Auckland. All tests were performed in accordance with the biomedical research guidelines for animal welfare and were approved by the University of Auckland committee for the ethical use of animals in scientific research (AEC# 2058, 2274, and 2148). All animal procedures performed were in accordance with the guidelines from Directive 2010/63/EU of the European Parliament on the protection of animals used for scientific purposes.

For whole-cell recordings of petrosal neurones, male Wistar and SH rats (*R. norvegicus*) were bred by the Animal Care Facility of the University of São Paulo, Campus of Ribeirão Preto, São Paulo, Brazil. All experiments complied with the Guide for the Care and Use of Laboratory Animals published by the Brazilian National Council for Animal Experimentation Control and with the Directive 2010/63/EU of the European Parliament on the protection of animals used for scientific purposes. The Institutional Ethics Committee approved all experimental protocols for Animal Experimentation at the School of Medicine of Ribeirão Preto/University of São Paulo (protocol 1/2016-1).

### 2.2 Working heart-brainstem preparation

**Figure 1:**
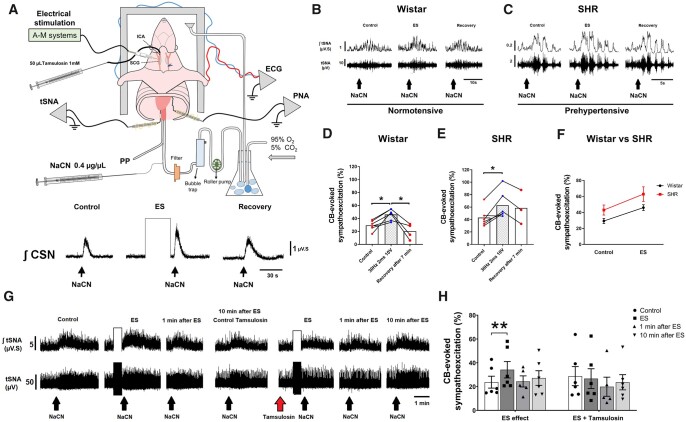
ES (30 Hz, 2 ms, 10 V) of the SCG leads to chemoreflex hyperreflexia of the sympathetic nerve response in both Wistar and SH rat (SHR) strains. (*A*) Schematic of the working heart-brainstem preparation (WHBP) showing cannulation of the ICA for drug infusions into the CB, and ES of SCG. Enhanced integrated CSN (∫CSN) discharge reveals a sensitizing effect of ES-SCG on the CB. (*B*) tSNA (raw and integrated waveform) during chemoreflex activation before and after ES in Wistar and (*C*) SHR. (*D*) Group data of ES of the SCG on the chemoreflex-evoked sympathoexcitation of Wistar (*n* = 6) vs. (*E*) SHR (*n* = 6). (*F*) The slope of linear regression between rat strains shows no difference in the gain of chemoreflex sensitization following ES of the SCG. (*G*) tSNA (raw and integrated waveform) during chemoreflex activation before and after ES and repeated after injection of tamsulosin (50 µL, 1 mmol/L) into ICA of a Wistar rat. (*H*) Tamsulosin blocked the sensitizing effect of ES-SCG on the CB-evoked sympathoexcitation (*n* = 6). Chemoreflex was evoked via intra-aorta injection of NaCN (0.4 µg/µL; 50 µL). Data analysed using paired Student’s *t*-test and mixed-effects model from ES onwards; *P < 0.05 and **P < 0.01. Note: the reduced number of data points in (*D* and *E*) reflects the loss of high-quality recordings in five preparations.

Juvenile Rats (3–6 weeks old, 50–90 g) were anaesthetized deeply with isoflurane (5% in O_2_, 1 L ${\text{min}}_{,}^{-1}$ via inhalation) until loss of paw withdrawal reflex, then given heparin intraperitoneally (350 UI, Pfizer, Australia). Subsequently, animals were euthanized via exsanguination through bisections below the diaphragm, and, after cooling the upper body in Ringer’s solution (composition in mmol/L as follows: NaCl, 125; NaHCO_3_, 24; KCl, 3.75; CaCl_2_, 2.5; MgSO_4_, 1.25; KH_2_PO_4_ 1.25; and D-glucose 10; Sigma-Aldrich, Australia), animals were decerebrated precollicularly. Lungs were removed, and the descending aorta was isolated and cannulated via a double-lumen catheter (Braintree Scientific, USA). Retrograde perfusion of the thorax and head restored viability based on the return of a ramp-like phrenic nerve (PN) pattern. The perfusate was Ringer’s solution containing an oncotic agent (1.5%, polyethylene glycol, 95172-250G-F, Sigma-Aldrich, Australia), gassed with carbogen (5% CO_2_, 95% O_2_), warmed to 31°C–32°C, filtered with nylon mesh (25 µm; Millipore) and recirculated. The second lumen of the cannula was connected to a Gould pressure transducer and amplifier (series 6600) to monitor perfusion pressure (PP) in the aorta (*Figure 1A*). The PP was held within 55–80 mmHg for Wistar and 70–90 mmHg for SH rats via the addition of vasopressin (Wistar: 2–2.5 nmol/L—SH rats: 3–3.5 nmol/L; V9879-5MG, Sigma-Aldrich, Australia) into the reservoir and adjusting the peristaltic pump flow (20–25 mL/min; Watson-Marlow 505 s, Falmouth, UK). Neuromuscular blockade was established using vecuronium bromide added into the reservoir 300 µL (10 mg/mL, Mylan, New Zealand). Recordings of PN, carotid sinus (CSN; identified as a branch of the glossopharyngeal nerve), and thoracic sympathetic nerve activity (tSNA; between T13 and L3) were obtained using bipolar glass suction electrodes. Signals were amplified (10 000×, A-M Systems model 1700), bandwidth filtered (10 Hz–1 kHz, A-M Systems), digitized (10 kHz, Micro1401-3, CED), and recorded using software Spike2 (CED). Average background tSNA noise was determined 15 min after the peristaltic pump was turned off, after brainstem death. Heart rate (HR) was derived from the inter R-wave of the electrocardiogram recorded through two electrodes and derived by using a window discriminator.

### 2.3 Peripheral chemoreflex—working heart-brainstem preparation

Sodium cyanide (NaCN; 50–100 µL; 0.4 µg/µL, Sigma-Aldrich, Australia) was injected as a bolus directly into the aorta from a pre-calibrated 100 µL Hamilton syringe to stimulate the CB chemoreceptors. The chemoreflex consisted of increased PN activity, bradycardia, sympathoexcitation, and an increase in PP. We quantified the chemoreflex in two ways: first, calculating the percentage increase in respiratory rate (i.e. tachypnoea) and sympathoexcitation relative to the baseline immediately before the stimulus; the period of baseline used for this calculation was of the same time-length as the chemoreflex response (e.g. 7 s). Second, the maximum bradycardia and increase in PP were calculated as the change (Δ) in HR (bpm) and PP (mmHg) relative to the baseline. Two consecutive chemoreflex responses of the same magnitude were obtained before initiating subsequent protocols (see below). At least 7 min were allowed to elapse between each NaCN dose.

### 2.4 Whole-cell recordings from petrosal ganglion chemoreceptive neurones

In the working heart-brainstem preparation (WHBP), the CB, CSN, and petrosal ganglion (PG) complex was isolated on the preparation’s right side. As described previously,^13^ we performed whole-cell patch-clamp recordings of chemoreceptive petrosal neurones with electrodes filled with a solution containing the following: (in mmol/L, 130 K-gluconate, 4.5 MgCl_2_; 14 tris-phosphocreatine, 10 N-(2-Hydroxyethyl)piperazine-N′-(2-ethanesulfonic acid) (HEPES); 5 Ethylene glycol-bis(2-aminoethylether)-N,N,N′,N′-tetraacetic acid (EGTA); 4 Na-ATP; 0.3 Guanosine 5′-triphosphate sodium salt hydrate (Na-GTP); pH 7.3, Sigma-Aldrich, Brazil). This solution had an osmolarity of ∼300 mOsmol/kg.H_2_O, and when filled the resistance of the tip ranged from 6 to 8 MΩ. Current-clamp recordings were performed with an Axopatch-200B integrating amplifier (Molecular Devices) and pClamp acquisition software (version 10.0, Molecular Devices). Gigaseals (>1 GΩ) were formed, and whole-cell configuration was obtained by suction. To enable stable whole-cell recordings, the PG was opened along its lateral aspect. A mesh grid was lowered onto the ganglion for stabilization, while permitting visualization of the PG. We used electrical stimulation (ES) of the CSN (the axons of petrosal neurones) to find the chemosensitive petrosal cells that were characterized functionally by their excitatory response to NaCN (50 µL; 0.3 µg/µL) injected into the aorta.

### 2.5 Telemetry instrumentation for *in vivo* BP recordings and chemoreflex testing

Under anaesthesia with isoflurane (2–5% in O_2_, 1 L min^−1^, via inhalation), adult male SH rats (30–34 weeks old, 300–350 g) were given single-abdominal subcutaneous injection of analgesic (0.05 mg/kg of Temgesic—buprenorphine—Indivior, Australia) and antibiotic (4 mg/kg of Baytril—enrofloxacin—Bayer Pharmaceuticals, Australia). Surgical fields were trimmed and disinfected using solutions of iodopovidone and chlorhexidine. Under aseptic techniques, a midline abdominal incision of 2.5 cm was made, and the descending abdominal aorta was exposed and dissected free of surrounding tissue. The aorta was briefly occluded, then pierced using a bent 23-G needle to help insert the BP catheter of the transmitter (TRM54P, Kaha Science, New Zealand). The catheter was advanced so that the tip was positioned just below the left renal artery. Blood flow was restored through the aorta once the probe was secured in place using tissue adhesive (VetBond, 3 M, USA) and polypropylene mesh (Small Parts Ltd, USA). The transmitter body was placed in the abdominal cavity and the abdominal muscle layer was closed with silk sutures.

After the BP telemeter was implanted, the right femoral vein was exposed via a 2 cm incision. The vein line was composed of two catheters of polyurethane connecting 3 cm of MRE-033 (Braintree Scientific, USA) pre-coated with heparin (TDMAC, Plolysciences, Eppelheim, Germany) with 16 cm of MRE040 (Braintree Scientific, USA). The line was pre-filled with locking solution (50 U/mL heparin + 2000 U/mL of penicillin G dissolved in sterile saline) and the catheter was inserted 1.5 cm into the femoral vein. The vein line was secured in place with tissue adhesive and polypropylene mesh. The catheter was tunnelled subcutaneously and connected to a capped intrascapular port. After the surgery, Temgesic (0.05 mg/kg—buprenorphine—Indivior, Australia) was given subcutaneously once a day for 2 days and the femoral line was flushed with heparinized saline solution every 2 days throughout the time of experiments. Animals were allowed a 7-day recovery period. After control data were obtained [i.e. pre-SCG resection (SCGx) chemoreflex response], a longitudinal incision was made on the ventral surface of the neck and the salivary glands, sternomastoid, and sternohyoid muscles gently separated and retracted, exposing the SCG. The ganglia were dissected free from connective tissue the vagus nerve and carotid arteries. Then, its connecting points with the cervical sympathetic trunk, external carotid nerve, and internal carotid nerve were severed. After removal of the ganglion, the neck incision was sutured, and animals were allowed a 5-day recovery period before re-testing the chemoreflex; Temgesic (0.05 mg/kg—buprenorphine—Indivior, Australia) was given subcutaneously once a day for 2 days post-SCGx. At the end of the experiments, animals were euthanized via intravenous injection of Pentobarb 300 (800 mg/kg—Sodium Pentobarbitone—Provet NZ Pty Ltd, New Zealand).

### 2.6 Peripheral chemoreflex—*in vivo*

The rats were challenged with potassium cyanide (KCN; 2 µg/µL, Sigma-Aldrich, Australia) injections, (i.v.) to evoke the chemoreflex. A dose-response curve was constructed for each animal with 4 doses—10, 20, 40, and 80 µg/rat regardless of body weight. Between each injection, we waited 15 min, so animals could recover their haemodynamic parameters to baseline levels. The maximum CB-evoked pressor and bradycardic responses were analysed after each KCN injection.

### 2.7 Experimental design

#### 2.7.1 *In situ* experiments

Six protocols were carried out using the WHBP to assess the modulatory effect of the sympathetic innervation on CB excitability. For all protocols in the WHBP, the left common carotid artery (CCA) was ligated to ensure only the CB chemoreceptors on the ipsilateral intervention side were stimulated. In protocols (ii), (iii), and (v) as described below, we cannulated the right internal carotid artery (ICA) with a fine cannula having a dead space of 10 µL, which was accounted for in all injections. The tip of this cannula pointed towards the CCA with its tip just rostral to the bifurcation and juxta-positioned to the CB artery; its other end was connected to a Hamilton syringe (100 µL). Proper position of the tip of the cannula close to the CB artery and the integrity of the CB and its CSN connection were confirmed by presence of the chemoreflex evoked by 20 µL of NaCN (0.4 µg/µL) locally injected into the ICA to stimulate the CB (see Supplementary material online, *Figure S1*, left panel). Before switching to different drugs, the Hamilton syringe was disconnected and perfusate permitted to flow through the catheter to rinse it out. Prior to the subsequent procedures detailed below, at least two consistent control chemoreflex responses were evoked by systemic injection of NaCN into the descending aorta in all cases.

##### 2.7.1.1 Stimulation of sympathetic efferent to the CB in Wistar and SH rats (n = 28)

In a pilot study, the right SCG of ten Wistar and six SH rats were isolated surgically, and a twisted wire bipolar microelectrode (wire diameters 0.125 and 0.150 mm, MS303-3B-SPC, PlasticsOne, USA) was placed onto the surface of the ganglion for ES (10–40 Hz, 0.1–2 ms, 5–10 V; A-M System isolated pulse stimulator Model 2100). The stimulating parameters were screened to generate the most consistent and reproducible CB-evoked responses. The stimulatory paradigm established was 30 Hz, 2 ms, and 10 V for 30 s. Once established, we carried out our protocol in six Wistar and six SH rats.

The dose of NaCN selected was sub-maximal but sufficient to produce measurable chemoreflex responses (i.e. when all CB-evoked cardiorespiratory motor outputs were present: increased CSN discharge, bradycardia, tachypnoea, and sympathoexcitation). Once selected, the dose was not changed throughout the experiment. Immediately after the ES was turned off, another dose of NaCN was injected to assess its effect upon the chemoreflex. These were classified into three categories: ‘attenuation’, ‘no effect’, and ‘sensitization’ based on either up— or downwards variation of ≥ 5% in the CB-evoked sympathoexcitation. As a control for stimulus spread, (i) we removed the SCG and electrically stimulated the exact same location (*n* = 2) and (ii) inactivated the ganglion with microinjection of lignocaine (1–2 µL, 2%; *n* = 5).

We used linear regression to fit the ES data for Wistar and SH rats where the slopes were used to compare the gain of sensitization between rat strains. In this protocol, for calculation of the chemoreflex outputs, the baseline used was an equivalent period before starting the ES.

##### 2.7.1.2 Blocking α_1_-adrenoreceptors prior to the ES of the SCG in Wistar rats (n = 6)

We repeated the previous protocol but now injecting an α_1_-adrenoreceptor antagonist to check whether this would prevent the ES sensitizing effect. First, we confirmed the ES was evoking a sensitizing effect and checked the chemoreflex recovery at 1min and 10 min after stimulation. Next, we injected tamsulosin (50 µL, 1 mmol/L in saline) into the ICA and repeated the ES.

##### 2.7.1.3 Blocking α_1_-adrenoreceptors in CBs of SH rats (n = 26)

We injected into the ICA either 40 µL of prazosin (1 mmol/L in saline pH = 3, i.e. 17 µg bolus, Sigma-Aldrich, Australia,—*n* = 10), an inverse agonist of α_1_-adrenoceptors, or 50 µL of tamsulosin (1 mmol/L in saline, i.e. 22 µg bolus, Tocris Bioscience, UK,—RDS305010—*n* = 6). In addition, the effect of prazosin on CSN discharge was also checked in six SH rats. Tamsulosin is a competitive antagonist of α_1_-adrenoceptors with greater selectivity to α_1A_ than α_1B_ receptor subtypes. Prazosin vehicle (saline pH = 3) was tested in four rats as a control.

##### 2.7.1.4 Activating α_1_-adrenoreceptors in CBs of Wistar rats (n = 13)

We injected 50 µL of phenylephrine (1 mmol/L in saline, i.e. 10 µg bolus; Sigma-Aldrich, Australia, *n* = 7) into the ICA to activate α_1_-adrenoceptors within the CB. The chemoreflex was re-assessed 20 s and 7 min after drug administration; the effect of phenylephrine on CSN discharge was also checked in six Wistar rats.

In our study, we used a mechanism of target engagement, where we aimed for a biological readout of drug effect. The dose was adjusted to produce a shift of at least 5 mmHg in PP in 5 min. Our rational was that if the amount of drug injected was able to produce changes in PP, it would be enough to evoke vascular response in the CB, thus altering its blood flow. The starting point for the dose of 1 mmol/L was based on previous study with phenylephrine.^27^

##### 2.7.1.5 Unilateral SCGx in SH rats (n = 16)

To test whether there was endogenous sympathetic tone modulating the CB in SH rats, we resected the right SCG (*n* = 10). The CB was repeatedly stimulated at 10, 17, and 25 min after SCGx. To confirm that our results were due to ablation of the SCG and not a loss of chemoreceptor sensitivity over time, we performed the same procedure in intact, non-ganglionectomized rats (*n* = 3). In addition, we also removed the SCG in Wistar rats (*n* = 3) to evaluate its role in normotensive animals.

##### 2.7.1.6 Whole-cell recordings of chemoreceptive petrosal neurones in SCGx SH rats (n = 15)

Blind whole-cell current-clamp recordings were carried out in five SCGx SH rats to compare their cellular electrophysiological properties against Wistar (*n* = 5) and non-ganglionectomized SH rats (*n* = 5).^13^ Depolarizing currents were injected to measure their neuronal excitability (0.5, 1.0, and 1.5 nA), whereas hyperpolarizing currents were injected to measure the neuronal input resistance (-0.5, −1.0, and −1.5 nA) and NaCN (0.3 µg/µL, 50 µL) was injected via aorta to compare the CB sensitivities.

#### 2.7.2 *In vivo* experiments

##### 2.7.2.1 Bilateral SCGx in adults SH rats in vivo (n = 5)

Finally, *in vivo* experiments were carried out to confirm the *in situ* results. BP telemeters were implanted in five adult SH rats to compare the CB excitability before and after bilateral SCGx and the long-term effects in BP. Rats underwent surgical implantation of a BP telemeter and femoral vein line as described above. Following 7 days of recovery, animals were challenged with KCN to evoke the chemoreflex. The latter was brought about in 2 consecutive days of testing, both before and after bilateral SCGx. On the first day, animals received increasing doses of KCN, i.e. 10, 20, 40, and 80 µg/rat. On the second day, the order was reversed (i.e. 80, 40, 20, and 10 µg). Next, we tested the chemoreflex sensitivity again on the 5th and 6th day post-SCGx using the same scheme of KCN doses described above. The chemoreflex response for each dose of KCN was average from both days pre-SCGx and both days post-SCGx. The data were collected by a blind investigator.

Four out of five rats were kept alive for 25 days for evaluation of long-term effects on BP and chemoreflex sensitivity. The chemoreflex was tested again on the 10th and 18th days after the surgery, with two rats receiving increasing doses of KCN and two receiving decreasing ones. The time-points for BP longitudinal analysis corresponded to epochs of 5 h collected from 17 to 22 h. Each BP telemeter’s offsets were measured before implantation and during post-mortem, then averaged and extracted from BP value at each time-point. The chemoreflex tests were always carried out between 10 and 13 h.

### 2.8 Immunohistochemistry

Sections of carotid artery bifurcations containing the CB were processed from three Wistar and three SH rats and stained for α_1A_- and α_1B_-adrenoceptors. The bifurcations were fixed in 4% paraformaldehyde in phosphate buffer saline (PBS; 0.1 mol/L, pH = 7.4,) overnight. Subsequently, sections were immersed in 20% sucrose for 24 h at 4°C, then embedded in OCT compound (Tissue-Tek^®^, PST-ProScitech, Australia), frozen, and stored at −80°C. The bifurcations were sectioned using a cryostat (10-µm thick) and mounted on glass slides (Superfrost^®^ Plus, LabServ, LBS4951+, New Zealand). Sections were permeabilized for 20 min (0.5% Triton X-100 in PBS), and blocked for 1 h in PBS-0.1% Tween20 (Sigma-Aldrich, Australia) containing 1% bovine serum albumin (BSA, pH Scientific Limited, New Zealand), 10% Donkey serum (Sigma-Aldrich, Australia), and 0.3 mol/L of Glycine (Sigma-Aldrich, Australia). Sections were incubated overnight in a humidified container at 4°C with primary antibodies (see Supplementary material online, *Table S1*). After washing, they were incubated for 2 h with secondary antibodies in PBS-0.1% Tween20 containing 1% BSA, 1% Donkey serum (see Supplementary material online, *Table S1*). Sections were mounted with anti-fade media [ProLong Glass Antifade Mountant with NucBlue (Hoechst 33342), Invitrogen; P36981, Thermofisher, New Zealand] and imaged using a confocal microscope (Zeiss LSM 800 Airyscan). Tyrosine hydroxylase (TH) staining was used as a marker for glomus cells, whilst α-smooth muscle actin (α-SMA) for contractile blood vessels. We performed negative control staining with secondary antibodies without the primary ones to exclude non-specific binding.

### 2.9 Data analysis

Nerve signals were rectified and integrated (□) with a time constant of 50 ms. Following prazosin injection, we assessed changes in the ongoing respiratory-sympathetic coupling, as this is important for the development and maintenance of hypertension.^28,29^ Data were averaged from epochs of 15 s collected from time-points prior to and after (i.e. 4 and 25 min) Prazosin injection. For analysis of respiratory-sympathetic coupling, we used a custom written analysis algorithm^30^ to detect each phase of the respiratory cycle. Expiratory (E) phases E1 and E2 were defined arithmetically and represent the first two-third and the final one-third of expiration, respectively. The maximum tSNA burst amplitude and the area under the curve (AUC) of each respiratory phase were calculated to quantify the respiratory-sympathetic coupling.

Statistical analyses were performed using GraphPad Prism (version 8.0, USA) and Statistical Package for the Social Sciences version 27.0 (SPSS, Chicago, IL, USA) softwares. Paired and unpaired Student’s *t*-test, as well as a repeated measure (RM) and ordinary one-way ANOVA were used accordingly. Due to the longevity of protocols, high-quality recordings could not be maintained in all preparations throughout the full extent of some studies; mixed-effects model was used instead of ANOVA if missing data were present in any group. To analyse the effects of unilateral SCGx on CB excitability of SH rats in the WHBP, we used the mixed-effects model. For these analyses, we incorporated the minutes 10, 17, and 25 post-SCGx as three levels within the factor ‘time’ and this was inserted as the within-subject effect and modelled using ‘AR1’ as the working correlation matrix (WCM). The factor ‘SCGx’ was added as a fixed factor whilst ‘control’ as the baseline covariate. Therefore, the model was equivalent to an RM two-way ANCOVA. To analyse the neuronal excitability in the whole-cell patch-clamp recordings, we used the generalized estimating equations (GEE) with a gamma distribution, which was chosen based on the Quasi Likelihood under Independence Model Criterion goodness of fit; The factor ‘depolarizing current’ was inserted as the within-subject effect and modelled using ‘independent’ as the WCM. For analysis of the chemoreflex *in vivo*, GEE were also used but with linear distribution. First, we evaluated which effect the SCGx would have on chemoreflex sensitivity. GEE was used to compare pre-SCGx vs. Day 5 post-SCGx with factors ‘Surgery’ and ‘KCN’ as within-subject effects and modelled using ‘independent’ as the WCM. Next, we analysed the effect of SCGx over time. For these analyses, we incorporated Days 5, 10, and 18 post-SCGx as three levels within the factor ‘time’; therefore, both factors ‘KCN’ and ‘time’ were modelled as within-subject effects using ‘unstructured’ as the WCM. The assumptions for each test were checked and when violated, a non-parametric test used, e.g. Wilcoxon test and Kruskal–Wallis. Dunnett’s *post hoc* test was used to adjust for multiple comparisons. For GEE analysis, we used Bonferroni *post hoc* test to adjust for multiple comparisons since Dunnett’s was not available in the software. Pearson correlation and linear regression analysis were used when necessary. The level of significance was set at *P* < 0.05 and data were expressed as mean ± standard errors of the mean.

## 3. Results

### 3.1 The SCG can sensitize CB chemoreflex

In a pilot study to determine the optimal parameters for ES of the SCG, we observed variable effects: ‘attenuation’, ‘no effect’, and ‘sensitization’ (see Data availability file). We quantitatively observed that higher voltages (8–10 V) and pulse widths of 2 ms tended to produce ‘sensitization’, as indicated by an increase in CB-evoked CSN activity (*Figure 1A*). Repeated stimulation of the SCG indicated that our stimulation protocol did not cause tissue degradation as the response was either not changed or showed mild sensitization (see Supplementary material online, *Figure S2*). Using this stimulus (i.e. 30 Hz, 2 ms, and 10 V), we found that the only component of the chemoreflex significantly enhanced was the sympathetic response. In Wistar rats (*n* = 6), the control response displayed an excitation of 29% ± 3.2% from baseline, whilst after ES this increased to 46% ± 3.6% [*Figure 1B and D*; *t*_(5)_ = 4.513; *P* = 0.006], which recovered to baseline levels after 10 min. ES of the SCG in SH rats (*n* = 6) further enhanced the CB-evoked sympatho-hyperreflexia [43% ± 6.3% vs. 63% ± 9.2%, *Figure 1C and E*; *t*_(5)_ = 3.631; *P* = 0.015]. The change in absolute sensitization was similar between rat strains (*Figure 1F*). ES-evoked CB sympatho-hyperreflexia was prevented by prior application of lignocaine injected into the SCG (*n* = 5, see Supplementary material online, *Figure S3*). ES in the locality of the SCG after its removal was also without effect (*n* = 2; data not shown), implying that the reflex sensitization was not due to stimulus spread to the other nearby structures, including the CB itself. In contrast, the CB-evoked bradycardia, tachypnoea, and pressor responses were all unchanged by ES of the SCG in both rat strains (see Supplementary material online, *Figure S4*).

Injecting tamsulosin (a competitive α_1_-adrenoreceptor antagonist) into the CB via the ICA prevented the sensitizing effect of ES of SCG on CB-evoked sympatho-hyperreflexia in Wistar rats (*Figure 1G and H*). The control response showed an excitation of 24% ± 5.0% from baseline, enhanced to 34% ± 6.9% [*t*_(5)_ = 4.685; *P* = 0.0027] by ES of the SCG, then returned to 27% ± 8.3% after tamsulosin (*Figure 1H*); a level not different from control.

### 3.2 Blocking α_1_-adrenoreceptors attenuates CB-evoked sympatho-hyperreflexia in SH rats

**Figure 2:**
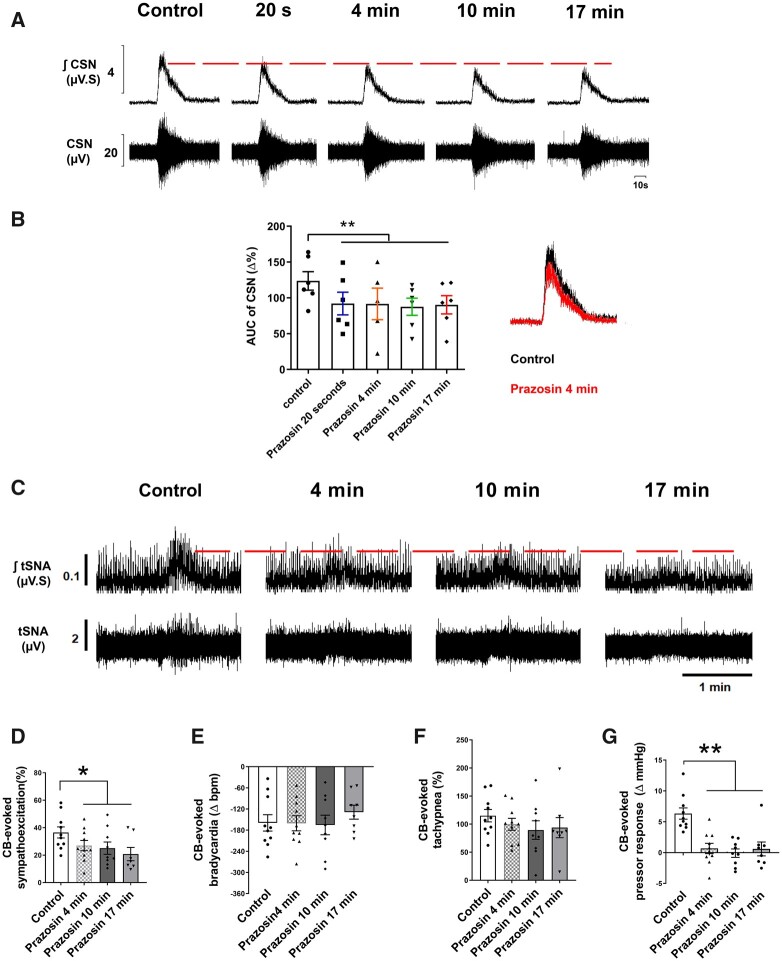
Prazosin injection (40 µL, 1 mmol/L) into the CB via the ICA attenuated the CB-evoked CSN discharge (*n* = 6), evoked sympatho-hyperreflexia and pressor response in SH rats (*n* = 10). (*A*) Typical tracing of CSN (raw and integrated waveforms) after chemoreflex stimulation with NaCN (0.4 µg/µL; 100 µL via aorta); on the right, the superimposition of control and prazosin responses after 4 min is shown. (*B*) A plot of the percentage change in the AUC relative to similar period of baseline. (*C*) Typical tracing of the tSNA (raw and integrated waveform) after chemoreflex stimulation. Other chemoreflex motor responses were not changed by prazosin. Group data for CB-evoked sympathoexcitation (*D*), bradycardia (*E*), tachypnoea (*F*), and pressor response (*G*) before and after prazosin. For CSN recordings, data were analysed using one-tail paired Student’s *t*-test, i.e. control vs. Prazosin 20 s and mixed-effects model from prazosin 20 s onwards, whereas for the CB-evoked motor responses, data were analysed using paired Student’s *t*-test or Wilcoxon test to compare control vs. Prazosin 4 min and mixed-effects model from prazosin 4 min; *P < 0.05 and **P < 0.01 vs. control. Note: the reduced number of data points in (*B*) reflects a missed injection for one time-point, whereas in (*D–G*), it reflects the loss of high-quality recordings in two preparations.

Injecting prazosin (an inverse agonist of α_1_-adrenoreceptors) into the CB via the ICA attenuated the CSN discharge 20 s after injection [*t*_(5)_ = 4.566, *P* = 0.003; *Figure 2A and B*]; no recovery was evident for 17 min. Likewise, prazosin attenuated the CB-evoked sympatho-hyperreflexia [from 37% ± 4.0% to 27% ± 3.7%; *Figure 2C and D*; *t*_(9)_ = 2.302; *P* = 0.0468] and the pressor response [from 6% ± 0.9% to 0.7% ± 0.8% mmHg; *t*_(9)_ = 6.225; *P* = 0.0002; *Figure 2G*] 4 min after the injection in SH rats showing no recovery thereafter. Regarding respiratory-sympathetic coupling, prazosin significantly reduced the resting tSNA peak-burst occurring at both the inspiratory (I) [25 min, *t*_(9)_ = 4.985; *P* = 0.0008] and post-inspiratory (E1) phases of the respiratory cycle [4 min, *t*_(9)_ = 2.448; *P* = 0.036; 25 min *t*_(9)_ = 9.230; *P* = 0.0001; see Supplementary material online, *Figure S5b* and *c*]. Furthermore, the AUC of tSNA during E1 and late expiratory (E2) phases were significantly reduced both at 4 [E1: *t*_(9)_ = 3.285; *P* = 0.0094, and E2: *t*_(9)_ = 2.441; *P* = 0.037] and 25 min [E1: *t*_(9)_ = 5.495; *P* = 0.0004, and E2: *t*_(9)_ = 4.332; *P* = 0.0019] after injection of prazosin (see Supplementary material online, *Figure S5f* and *g*). ICA injections of vehicle had no effect on the chemoreflex response (see Supplementary material online, *Figure S6*).

We next blocked α_1_-adrenoreceptors with ICA injections of tamsulosin, which attenuated the CB-evoked sympatho-hyperreflexia in SH rats [from 74% ± 10.95% to 52% ± 9.15%; Supplementary material online, *Figure S7a* and *b*; *t*_(5)_ = 4.374; *P* = 0.0072]. Similar to prazosin, we did not see a recovery of the effect over the time course studied [*F* (_1.707, 8.537_) = 3.885; *P* = 0.067]. This protocol was not performed in Wistar rats, which do not show CB-evoked sympatho-hyperreflexia.

### 3.3 Activating CB α_1_-adrenoreceptors in Wistar rats sensitizes chemoreflex-evoked sympathoexcitation

Phenylephrine injection into the CB via the ICA sensitized the CB-evoked sympathoexcitation in Wistar rats. The control response was enhanced from 27% ± 6.4% to 36% ± 7.6% [see Supplementary material online, *Figure S8a* and *b*; *t*_(6)_ = 3.561; *P* = 0.012]. We noted that this sensitized response was not different from the control response in SH rats [43% ± 6.3% vs. 36% ± 7.6%; *t*_(11)_ = 0.6683; *P* = 0.5177]. Seven min after phenylephrine, the CB-evoked sympatho-hyperreflexia had recovered to control levels (26% ± 2.9%; see Supplementary material online, *Figure S7b*). Phenylephrine injections raised baseline PP (see Supplementary material online, *Figure S8a*), potentially causing a baroreflex-mediated inhibition of tSNA (see Supplementary material online, *Figure S9*). We tested the possibility that baroreflex activation was tempering the sensitization of the chemoreflex. We correlated baroreflex mediated sympathetic inhibition with the CB-evoked sympatho-hyperreflexia after phenylephrine injection (see Supplementary material online, *Figure S10*) and found no correlation (*r* = −0.438; *P* = 0.384) suggesting an absence of any interaction. Phenylephrine also sensitized the CSN discharge 20 s after injection [*t*_(5)_ = 2.294, *P* = 0.035]; differently though, it did not return to control levels (see Supplementary material online, *Figure S11*).

### 3.4 Ablating the sympathetic innervation of the CB attenuates chemoreflex hypersensitivity in SH rats

**Figure 3:**
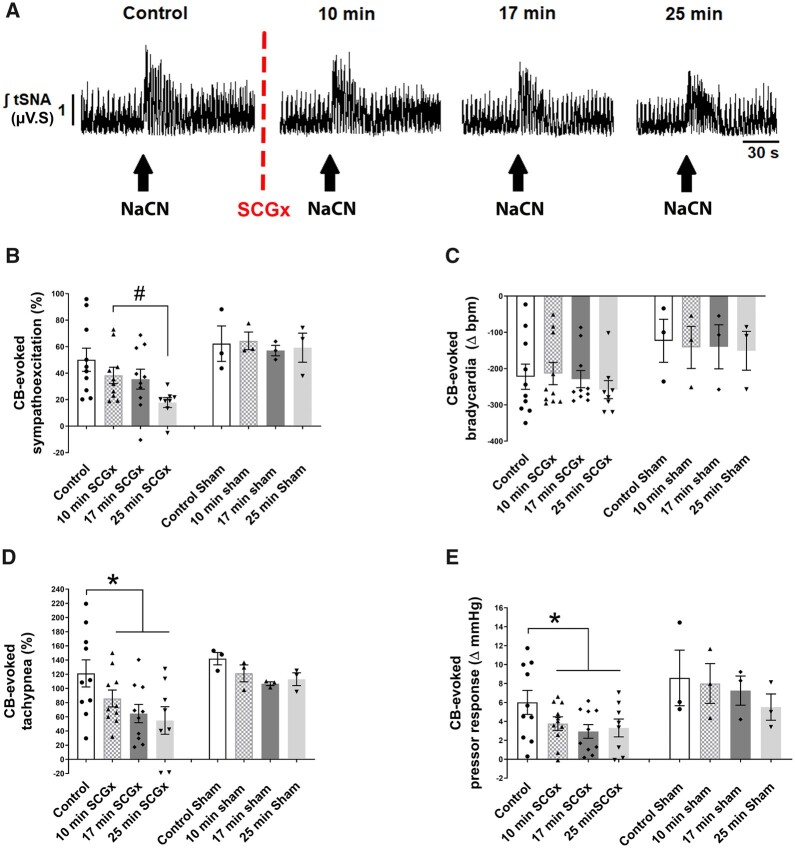
Unilateral SCGx attenuates the ipsilateral CB-evoked sympathoexcitation (*A* and *B*), tachypnoea (*D*), and pressor responses (*E*) in SH rats (*n* = 10). (*C*) the CB-evoked bradycardia was unchanged. (*A*) Typical tracing of the tSNA (raw and integrated waveform) after chemoreflex stimulation. Sham (SCG) SH rats (*n* = 3) do not show a change in chemoreflex response over time. The chemoreflex was evoked with NaCN (0.4 µg/µL; 100 µL). Data were analysed using paired Student’s *t*-test to compare control vs. 10 min SCGx and a mixed-effects model from post 10 min SCGx onwards; *P < 0.05 vs. control and #*P* < 0.05 vs. 10 min ganglionectomy. Note: the reduced number of data points in (*B–E*) reflects the loss of high-quality recordings in two preparations.

Unilateral (right side) SCGx attenuated the ipsilaterally CB-evoked responses in SH rats (*Figure 3A*). Whereas the respiratory response only showed a marginal *P* value [*F*_(1, 9.4)_ = 4.395; *P* = 0.064], both sympathetic [*F*_(1, 11.72)_ = 10.32; *P* = 0.008], and pressor responses [*F*_(1, 10.21)_ = 5.567; *P* = 0.038] showed time-dependent attenuation. CB-evoked sympatho-hyperreflexia was reduced 25 min (*P* = 0.0120; *Figure 3B*) after SCGx, whereas the CB-evoked tachypnoea [*Figure 3D*; *t*_(9)_ = 2.590; *P* = 0.0292] and pressor responses [*Figure 3E*; *t*_(9)_ = 2.768; *P* = 0.0218] were already reduced 10 min thereafter. The CB-evoked bradycardia (*Figure 3C*) remained unchanged. In contrast, in Wistar rats, unilateral SCGx did not change CB-evoked sympathoexcitation (see Supplementary material online, *Figure S12*). After unilateral SCGx, in both strains the resting PN rate was increased [SH rats, from 18 ± 1.9 to 23 ± 2.4 burst/min Wilcoxon matched-pairs signed rank test *W* = 55.00; *P* = 0.002; Wistar from 18 ± 3.0 to 25 ± 2.8 burst/min, *t*_(4)_ = 4.842; *P* = 0.0084], whilst the maximum CB-evoked tachypnoea was unchanged (data not shown). In contrast to PN frequency, unilateral SCGx did not change resting levels of tSNA in either strain (data not shown).

### 3.5 SCG ablation eliminates the hyperexcitability of chemoreceptive PG neurones

**Figure 4:**
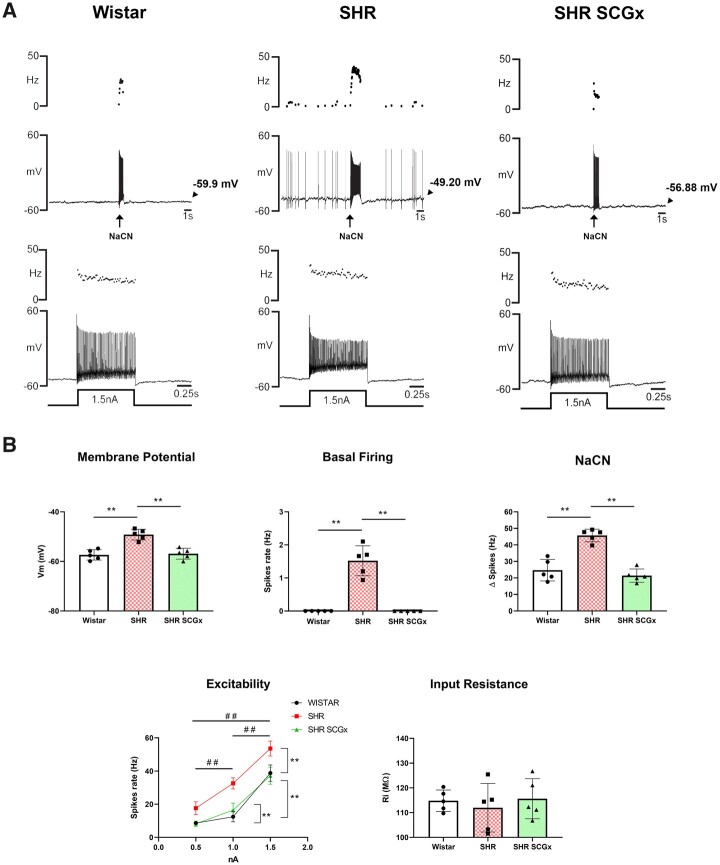
Effect of unilateral SCGx on ipsilateral chemoreceptive PG neurones. (*A*) Representative whole-cell current clamp recordings from chemoreceptive petrosal neurones from a Wistar (top left, *n* = 5), SH rat (SHR; top middle, *n* = 5), and SCGx SHR (top right, *n* = 5) recorded from the *in situ* WHBP. Ongoing and evoked firing responses to chemoreflex stimulation or injected depolarizing current were all reduced after SCGx in SHR. (*B*) Changes in resting membrane potential (*V*_m_), spontaneous basal firing, reflex responsiveness to NaCN injections (50 µL of 0.3 µg/µL), input resistance, and firing rate to injected depolarizing currents (0.5, 1, and 1.5 nA) are shown. Data were analysed using one-way ANOVA with Dunnett’s *post hoc* test or Kruskal–Wallis with Dunn’s *post hoc* test. Neuronal excitability was analysed using GEE and Bonferroni as *post hoc* test; *P < 0.05; **P < 0.01; ^##^*P* < 0.01 vs. injected current.

Unilateral SCGx in SH rats markedly reduced the electrical excitability of chemoreceptive petrosal neurones (*Figure 4*). Resting membrane potential (*V*_m_) was hyperpolarized [*F*_(2, 12)_ = 22.68, *P* < 0.0001] from −49 ± 0.96 to −56 ± 0.97 mV, which was not different from the level seen in Wistar rats. Spontaneous basal firing was eliminated by SCGx in SH rats (Kruskal–Wallis statistic = 13.29, *P* = 0.001). Further, there was an attenuation of the firing response evoked by NaCN injections [*F*_(2, 12)_ = 35.64, *P* < 0.0001] that was similar in magnitude to that observed in Wistar rats. The firing response to injection of depolarizing current was reduced by SCGx in SH rats [Wald χ^2^_(2)_ = 263.085, *P* ≤ 0.0001] to a level seen in Wistar rats. Input resistance was not different between Wistar, SH, or SH rats after SCGx [*F*_(2, 12)_ = 0.3032, *P* = 0.744].

### 3.6 SCGx attenuated the evoked chemoreflex response and reduced BP in *in vivo* conscious SH rats

**Figure 5:**
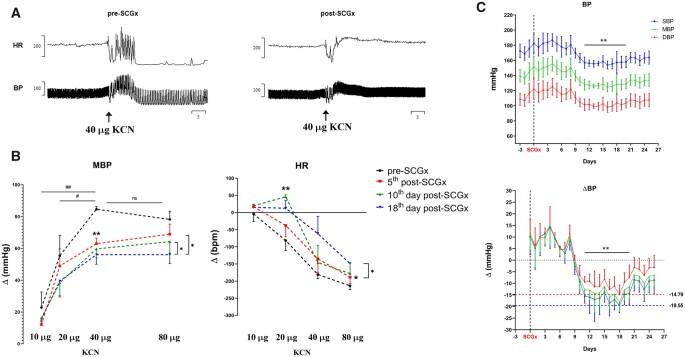
Effects of bilateral resection of the SCGx on chemoreflex and resting BP in *in vivo* conscious SH rats. The chemoreflex was evoked by intravenous injections of KCN (2 µg/µL; *n* = 5). The depicted responses for each dose of KCN represent the average from 2 consecutive days from both pre-SCGx and post-SCGx (i.e. 5th and 6th day after ganglionectomy). (*A*) Representative tracing from the CB-evoked cardiovascular response (KCN = 40 µg) before and after bilateral SCGx. (*B*) CB-evoked changes in MBP, mean blood pressure; HR, heart rate. (*C*) Long-term effect of bilateral SCGx on BP of SH rats (*n* = 4). BPs have averaged epochs of 5 h recordings performed between 17 and 22 h. Data analysed using GEE and Bonferroni as *post hoc* test; *P < 0.05; **P < 0.01; ^##^*P* < 0.01. Mixed-effects model was used to detect changes in BP over time; Dunnett’s *post hoc* test was used to compare post-SCGx days vs. Day 0, i.e. SCGx, **P < 0.01.

Bilateral SCGx in adult SH rats attenuated the CB-evoked pressor [Wald χ^2^_(1)_ = 7.563, *P* = 0.006] and bradycardic [Wald χ^2^_(1)_ = 11.713, *P* = 0.001] responses. Chemoreflex was tested 5 days post-SCGx (*Figure 5A and B*), which was further attenuated 2 weeks later [Hypertensive—KCN*time, Wald χ^2^_(4)_ = 12.032, *P* < 0.017; Bradycardia—KCN*time, Wald χ^2^_(4)_ = 242.699, *P* < 0.0001]. Eleven days after the SCGx, a significant fall in SBP [*F*_(25, 74)_ = 4.775, *P* < 0.0001] and DBP [*F*_(25, 74)_ = 3.810, *P* < 0.0001] was observed (*Figure 5C*). SBP and DBP fell on average 16 ± 4.85 and 10 ± 3.81 mmHg, respectively, reaching a nadir of −19.5 and −14.8 mmHg on the 18th day post-SCGx. From the 20th day onwards, BP gradually increased, although it had not recovered to pre-SCGx levels by the end of the protocol on Day 25.

### 3.7 Immunohistochemistry

**Figure 6:**
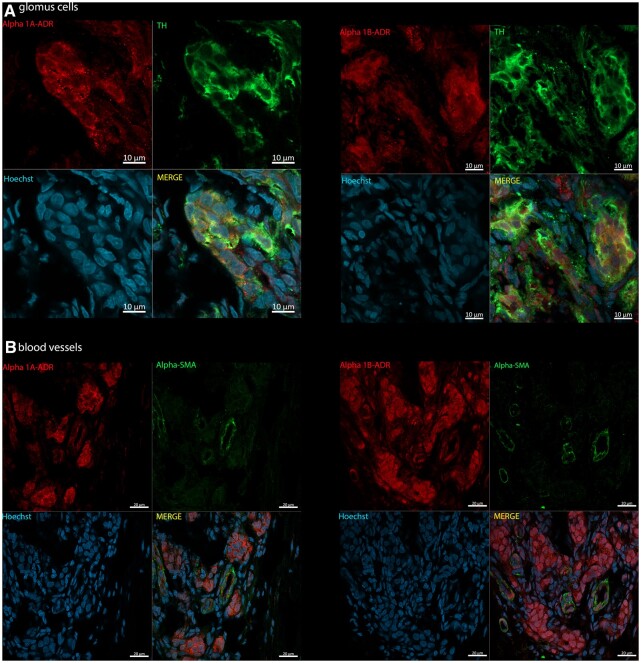
Photomicrographs of CB immunopositivity for TH and α_1_-adrenoceptor subtypes in Wistar rats (*n* = 3). (*A*) α_1A_-and α_1B_-adrenoreceptors (Alpha 1A/B-ADR; red, Alexa Fluor 594) with TH (green, Alexa Fluor 488), a marker for glomus cells. (*B*) α_1A_- and α_1B_-adrenoreceptors (Alpha 1A/B-ADR; red, Alexa Fluor 594) co-localized with α-SMA (green, Alexa Fluor plus 488), a marker for blood vessels. Hoechst staining is shown for cellular nuclei (blue). Images acquired using Zeiss LSM 800 Airyscan.

*Figure 6* depicts immunofluorescence for both α_1A_- and α_1B_-adrenoreceptors within the CB of Wistar rats. We found both α_1_-adrenoreceptor subtypes expressed on glomus cells (i.e. positive TH, Green; *Figure 6A*). Some blood vessels also expressed α_1A_- and α_1B_-adrenoreceptors (*Figure 6B*, positive α-SMA, Green). The presence of both sub-types was observed in the same vessel. α_1_-adrenoreceptors were equally expressed in glomus cells and blood vessels of SH rats (see Supplementary material online, *Figure S13*). Negative control staining showed no non-specific staining from secondary antibodies (see Supplementary material online, *Figures S14* and *S15*).

## 4. Discussion

Our findings provide evidence that the SCG, the key source of sympathetic innervation to the CB, mediates hypertonicity and increased activity of the arterial chemoreflex evoked from the CB in an animal model of essential hypertension. Specifically, our data reveal that sympathetic activity evoked from the SCG by ES causes CB sympatho-hyperreflexia in Wistar and SH rats. This sensitization was restricted to the sympathetic component of the chemoreflex response, with a similar magnitude of effect between rat strains, and mediated by α_1_-adrenoreceptors as it was prevented by tamsulosin. We found that the chemoreflex can also be sensitized by phenylephrine (an α_1_-adrenoreceptor agonist) when applied to the CB of normotensive Wistar rats. However, only in SH rats was the sympathetically mediated CB sensitization tonically active as revealed by SCGx that attenuated CB-evoked sympatho-hyperreflexia and reset the electrical excitability of chemoreceptive petrosal neurones to levels observed in Wistar rats. Taken together, these data indicate a tonically active drive from the SCG that boosts CB sensitization in SH rats to explain their CB hyperreflexia.

As recently reviewed, studies investigating how the autonomic nervous system modulates CB excitability have been carried out since the 1950s.^25^ ES of the sympathetic innervation to the CB in normotensive animals (typically anaesthetized cats) was first performed by Floyd and Neil^23^ who, among others, showed that these fibres are able to increase the chemo-afferent firing rate.^23,31,32^ However, our study is the first to evaluate the functional effect of sympathetic activity on CB-evoked reflex responses and its tonicity in hypertension.

The presence of endogenous sympathetic modulation of CB function in SH rats is supported by our findings that (i) both prazosin and tamsulosin injected into the CB via the ICA reduced CB-evoked sympatho-hyperreflexia *in situ*, which was mimicked by SCGx; (ii) bilateral SCGx in conscious *in vivo* SH rats attenuated the hypertensive and bradycardic responses to chemoreflex activation chronically; (iii) the presence of α_1A_- and α_1B_-adrenoreceptors on both glomus cells and contractile blood vessels; thus, the cellular origin/s mediating the sensitization remains elusive.

**Figure 7:**
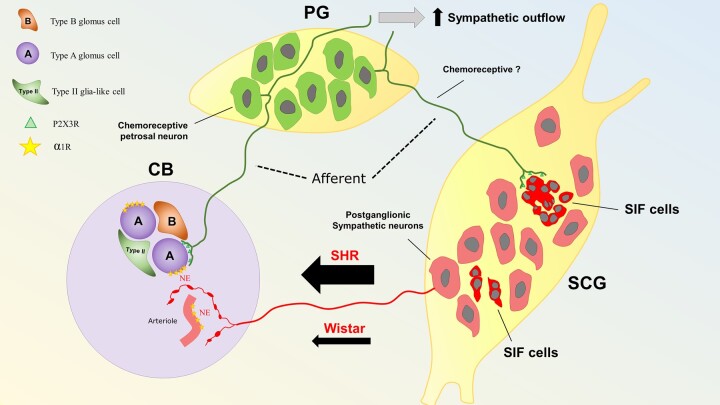
Proposed mechanism of hyperexcitability of the CB in SHR. The CB receives afferent (green) and efferent (red) innervation from the PG and SCG, respectively. Within the CB, connections are made with two main structures: Type A glomus cells and vasculature (arterioles, capillaries, and venules). Type A glomus cells receive inputs from purinergic PG afferent neurones positive for P2X3 receptors (P2X3R).^13^ In contrast, the vasculature is innervated by postganglionic sympathetic neurones (red) from the SCG. Both the CB vasculature and glomus cells express α_1_-adrenoreceptors (α1R; *Figure 6*) and although glomus cells are not directly innervated by postganglionic sympathetic neurones, sympathetic varicosities were shown to be located close enough to glomus cells clusters to allow noradrenaline (NE) to diffuse and reach their receptors. In SHR, a more active sympathetic input to the CB exists compared with Wistar rats (*Figures 3–5*). In the SCG, a subpopulation of SIF (intense red) cells, organized in clusters, receive innervation from afferent purinergic PG neurones; these cells have been proposed to be ectopic glomus cells and could contribute to the CB-evoked sympatho-hyperreflexia and increased peripheral sympathetic outflow. Data from.^25,34,38,39^

An intriguing observation was a predominant effect of the SCG on the chemoreflex-evoked sympathetic response. Although we do not fully understand the basis for this selectivity, we suggest that it relates to the distinct connectivity between subsets of glomus cells and reflex pathways likened recently to a ribbon cable or ‘private lines’ of communication.^7^ Thus, efferent modulation of the CB by the SCG would augment a subset of glomus cells controlling sympathetic motor activity. This suggests the existence of intricate inter-connections between the SCG and CB. In this context, retrograde labelling studies indicate that the SCG receives innervation from the PG.^33,34^ Approximately half of these PG fibres terminating in the SCG express purinergic P2X3 receptors; the latter make sensory afferent synapses with small intensely fluorescent (SIF) cells. The SIF cells modulate activity of both pre-and postganglionic neurones of the SCG^35^ and appear to be involved in the upregulation of norepinephrine synthesis in SCG postganglionic neurones in response to hypoxia.^36^ The SCG afferents expressing P2X3 receptors making connection to SCG SIF cells hold striking alignment to the CB’s glomus cells making synaptic contact with purinergic PG fibres that are distinctly and uniquely involved in the sympathoexcitatory component of the chemoreflex in SH rats^7,13,37^ (*Figure 7*). Indeed, it was proposed that the SIF cells receiving PG P2X3 receptor afferent fibres are ectopic glomus cells.^34^ Although the chemosensitivity of PG neurones supplying the SCG has not been determined, it is tempting to speculate that these purinergic projections from the PG to the SCG function as a feed-forward control that selectively sensitizes the sympathoexcitatory output from the CB upon stimulation of the SCG. This might explain how it is possible to modulate one component of the chemoreflex but not another.

Our finding of endogenous modulation by the SCG on CB sensitivity in SH but not Wistar rats is consistent with the hyperexcitability of both the sympathetic nervous system and the CB in this rat model.^13^ At the level of single chemoreceptive PG neurones, SCGx caused hyperpolarization, abolished basal neuronal firing, and reduced the CB-evoked firing response to levels found in Wistar rats. These data demonstrate that the SCG is driving CB hyperexcitability in SH rats. Of note was the 25 min delay from the time of unilateral SCGx to the attenuation of the chemoreflex sympathoexcitatory response. We propose that this reflects time needed to reduce long-term potentiation (LTP) of the CB induced by the SCG presumably involving second messenger systems. Of note is the presence of LTP in the SCG of SH rats which is present at their pre-hypertensive age^39–41^; whether the CB shares this property remains to be validated.

Considering the potential mechanism underlying the sympathetic modulation of CB excitability, one possibility is that a change in the chemoreflex gain is mediated by vasoconstriction of the CB vasculature to reduce its blood flow. This hypothesis is supported by the contrasting actions of α_1_-adrenoreceptor agonists and antagonists that increased and decreased CB-evoked sympatho-hyperreflexia as well as the CSN discharge, respectively. Winder *et al*.^42^ were the first to report excitation of CB-evoked chemosensory afferent discharge by reducing its blood supply. Later, Daly *et al*.^32^ showed that activation of CB sympathetic efferent fibres reduced CB blood flow, with an associated increase in excitability. More recently, Ding *et al*.^19^ proposed that in heart failure with reduced ejection fraction, acute falls in cardiac output lowers the blood flow to the CB, thus triggering hyperactivity. We hypothesize that the prevailing increased levels of sympathetic activity in SH rats^29^ result in reduced CB blood flow, driving its sensitization and subsequent chemoreflex hyperreflexia. Our immunolabelling of α_1_-adrenoceptors on CB vessels provides evidence that the necessary vascular signalling pathway is present. However, we acknowledge the evidence from ourselves (*Figure 6*) and others^18,21,26,27^ indicating that there is also extensive autonomic efferent innervation of non-vascular tissues in the CB. To what extent the vascular vs. non-vascular α_1_-adrenoceptors modulate CB chemoreceptors is still a matter of debate^28^ and requires further study.

In our experiments with phenylephrine injection, we observed a discrepancy between the results of CSN recordings and sympathetic motor output; whilst in the former, the CSN discharge did not return to baseline levels after 7 min, such a recovery was observed for CB-evoked sympathoexcitation. We do not fully understand this discrepancy, but it should be acknowledge that many factors can interplay with peripheral chemoreceptors on the respiratory-cardiovascular system integration to yield any motor response.^26^ Therefore, despite the fact of chemoreceptors being sensitized, this not necessarily means that such sensitization will always be translated into an increased reflex tSNA response.

A previous study found that in C57BL6 mice^43,44^ the SCG provided an inhibitory input to the CB. The removal of the cervical sympathetic chain in these animals led to increased ventilatory responses to hypoxic gas challenge. This effect may not necessarily be due to changes in CB excitability. A connection exists between the SCG and the nodose ganglion in C57BL/6J mice^45^ and in about 40% of rats^33,46,47^. Such connectivity is present between postganglionic sympathetic and vagal afferent neurones. Given this, it is plausible that the SCG provides excitatory input to vagal sensory neurones that depress ventilation such as those mediating pulmonary J and stretch receptor reflexes. Thus, the SCG may indirectly facilitate inhibitory inputs to brainstem inspiratory neurones whose activities are abolished once the SCG is removed. This is consistent with the raised PN rate we found after SCGx. One might argue that in the study of Getsy *et al*.,^44^ the basal ventilatory parameters are not different between sham and SCGx mice; however, these parameters were recorded only 4 days after SCGx and compensatory mechanisms may have occurred.

We have demonstrated that sympathetic efferents can affect chemoreceptor sensitivity. This mechanism is mediated via α_1_-adrenoreceptors and activated tonically in hypertension. We propose this effect may be due to sympathetically mediated reductions in CB blood flow; however, we cannot rule out a contribution from non-vascular pathways, since glomus cells were also positive for α_1_-adrenergic receptors. In our study, we did not measure the BP of Juvenile Wistar and SH rats and this is a limitation we acknowledge. However, it is well documented that by the age of 6 weeks old, SH rats have SBP around 110 mmHg, which is not significantly different from control rats (i.e. 100 mmHg) thus being considered as prehypertensive.^39^ Although juvenile SH rats have not developed their BP phenotype, previous studies have shown that these rats have overactivity of sympathetic outflow and CB hyperexcitability,^13,29^ which is what we wish to control thereby making this age group a most suitable model to investigate CB pathophysiology in hypertension. Given that reduced CB hyperexcitability is associated with falls in arterial pressure in hypertension^13,15,48^ and that the SCG provides the dominant innervation of the CB, we propose that the SCG may be a viable target for treating CB pathophysiology in hypertension.

We acknowledge that, in the future, further experiments need to be carried out on different models of hypertension, such as inducible model, which may further support the SCG as a viable target. Our results in *Figure 3* show a reduced CB-evoked tachypnoea, which could indicate that therapeutic ablation of SCG might interfere with physiological response of CB to hypoxia and might compromise ventilatory responses to hypoxia/high altitude. Unfortunately, we did not have the means to measure the respiratory response in our chronic *in vivo* protocol; however, as mentioned before, the reduced tachypnoea was due to an increase in basal PN rate induced by unilateral SCGx whereas the peak of the tachypnoeic response was unchanged. Furthermore, Getsy *et al.*^44^ did not report impaired ventilatory responses to hypoxic gas challenge in bilaterally SCGx mice. Therefore, we do not believe the ablation of SCG would chronically interfere with CB ventilatory response to hypoxia consistent with our finding of differential sympathetic modulation.

## 5. Translational perspective

We believe that severing the main connection between the SCG and CB (i.e. the ganglioglomerular nerve^25^) is a potential translational approach for normalizing CB sensitivity and as a hypertension treatment. Unilateral resection of the CB was demonstrated to reduce the BP in patients with resistant hypertension^15^; however, safety concerns about removing the CB and its control of ventilation need to be acknowledged.^49^ Selective denervation of the ganglioglomerular nerve in humans would mean that both the CB and SCG maintain their physiological function thereby minimizing any side effects. To our knowledge, this has never been performed previously and awaits trialling.

In our *in vivo* protocol, following bilateral SCGx the reduction in BP lessened somewhat after 18 days suggesting partial compensation. Thus, this may minimize the long-term clinical impact of SCG-targeted interventions. However, at the end of our protocol, the SBP was still reduced relative to pre-SCGx levels. The nadir for SBP was −19 mmHg (Day 18), whereas from the Day 21 onwards, the SBP stabilized to −10 mmHg. Meta-analyses of clinical trials have demonstrated that reductions of 10 mmHg in SBP were associated with reduced risk to stroke, coronary events, and heart failure (35%, 20%, and 40%, respectively) as well as a reduction in all-cause mortality to cardiovascular diseases (10–15%).^50,51^ In this regard, studies to evaluate BP over months following SCGx would be of great value to further assess the feasibility of the SCG as a therapeutic target.

## Supplementary material

Supplementary material is available at *Cardiovascular Research* online.

## Supplementary Material

cvac008_Supplementary_Data

## Data Availability

The authors declare that all supporting data have been made publicly available at the Figshare and can be accessed via doi (https://doi.org/10.6084/m9.figshare.14832759). Raw data are available from the corresponding author upon reasonable request.
