# Supplementary material for: The sympathetic nervous system exacerbates carotid body sensitivity in hypertension
Source: Cardiovasc Res. 2022 Jan 20;119(1):316–31. doi: 10.1093/cvr/cvac008 (PMC10022867; doi:10.1093/cvr/cvac008)
Supplement: cvac008_Supplementary_Data [file cvac008_supplementary_data.docx]

**DATA SUPPLEMENT**

**The sympathetic nervous system exacerbates carotid body sensitivity in hypertension**

Igor S. A. Felippe^1^, Tymoteusz Zera^2^, Melina P. da Silva^3^, Davi J. A. Moraes^3^, Fiona McBryde^1^, Julian F. R. Paton^1^

*^1^Department of Physiology, Manaaki Mānawa – The Centre for Heart Research, Faculty of Health & Medical Sciences, University of Auckland, Grafton Campus, Auckland, 1023, New Zealand.*

*^2^Department of Experimental and Clinical Physiology, Laboratory of Centre for Preclinical Research, Medical University of Warsaw, Warsaw, Poland*

*^3^Department of Physiology, School of Medicine of Ribeirão Preto, University of São Paulo, Ribeirão Preto, São Paulo, Brazil;*

**Supplementary figures/tables**


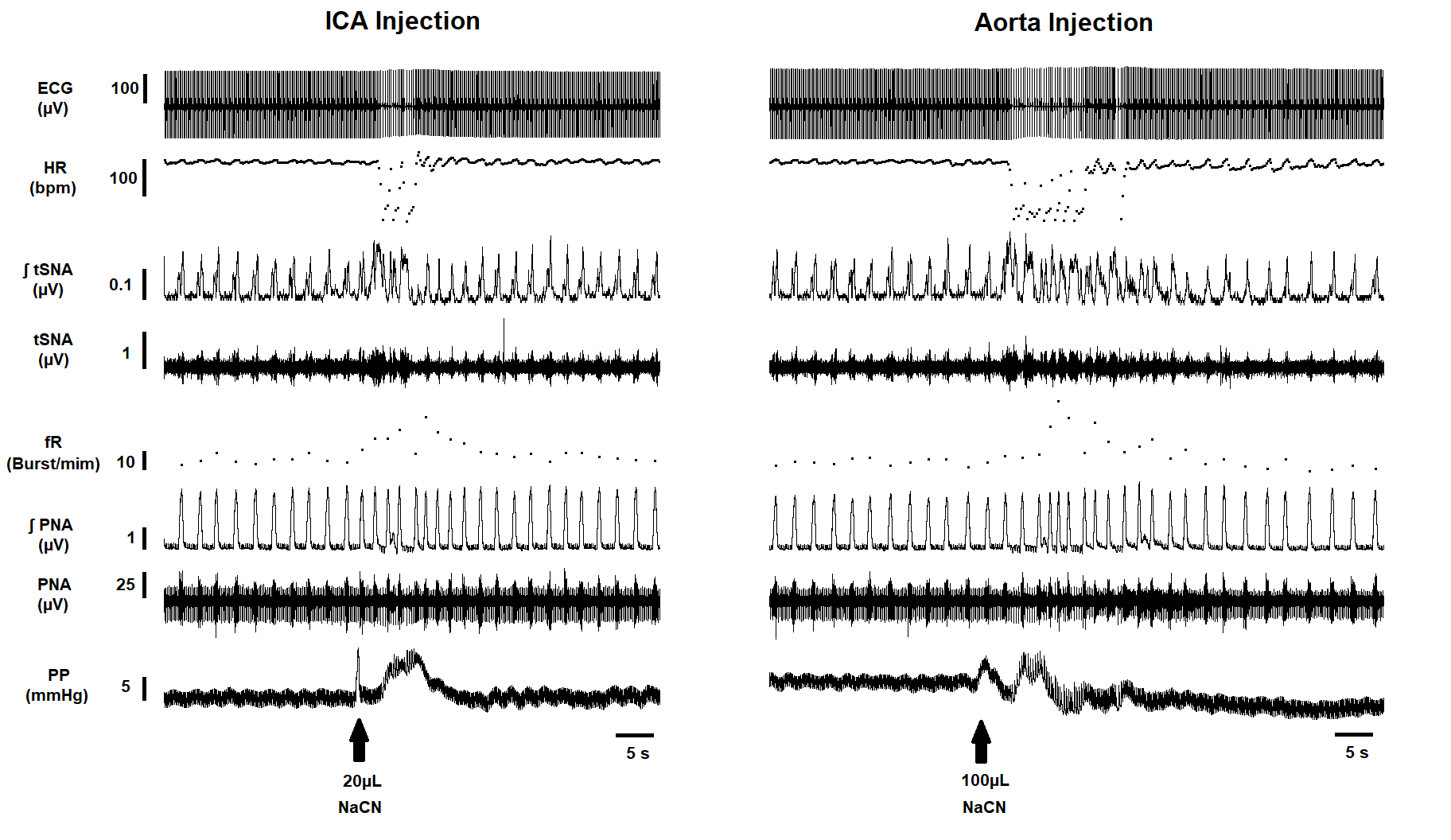


**Figure S1:** Routes of sodium cyanide (NaCN) administration in a spontaneous hypertensive rat. NaCN (0.4 µg/µL) was injected via the internal carotid artery (ICA, panel left) or the descending aorta (panel right) to evoke chemoreflex motor output responses before left carotid artery ligation.


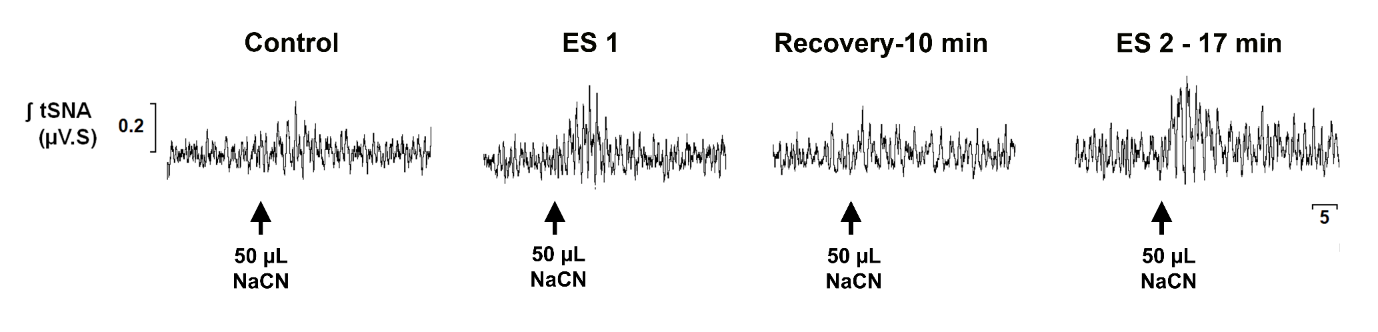


**Figure S2:** Repetitive electrical stimulation (ES) of the superior cervical ganglion. In a pilot study, stimulating the SCG of a Wistar rat showed that a second stimulation was still capable of sensitizing the CB-evoked sympathoexcitation (NaCN, 0.04%; 50 µL), indicating that our stimulation protocol did not cause non-specific tissue degradation of the SCG.


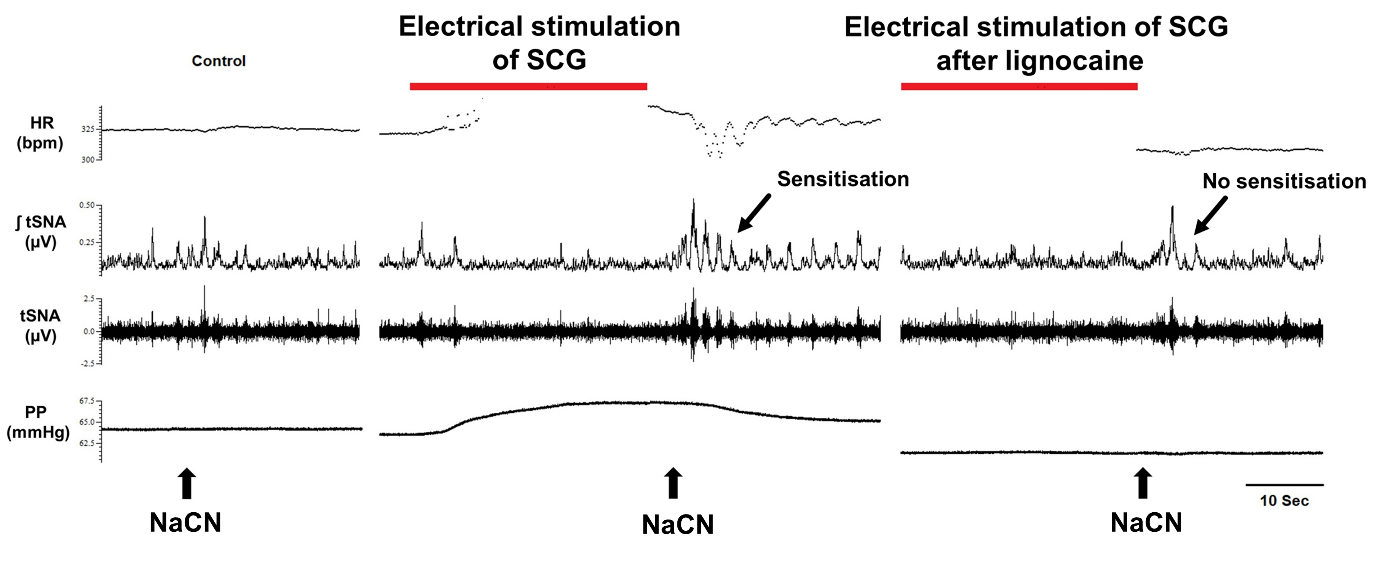


**Figure S3:** Inactivation of the superior cervical ganglion (SCG) with microinjection of 1-2 µL of lignocaine (2%) prevents sensitisation of chemoreflex sympathoexcitation in a spontaneous hypertensive rat. Chemoreflex was activated with sodium cyanide (NaCN 0.4 µg/µL; 50-70 µL).

**Table S1**: Primary and secondary antibodies for Immunohistochemistry

| **Class** | **Host** | **Immunogenicity** | **Titration** | **Company** | **Catalogue number** |
| --- | --- | --- | --- | --- | --- |
| **Polyclonal** | Chicken | Tyrosine hydroxylase (TH) | 1:50 | Abcam | ab76442 |
| **Polyclonal** | Rabbit | α_1A_-adrenergic receptors | 1:100 | Invitrogen | PA1047 |
| **Polyclonal** | Rabbit | α_1B_-adrenergic receptors | 1:50 | Invitrogen | PA526411 |
| **Polyclonal** | Goat | α-smooth muscle actin (α-SMA) | 1:100 | Invitrogen | PA5-18292 |
|  |  |  |  |  |  |
| **Polyclonal** | Donkey | Anti-chicken Alexa Fluor 488 | 1:200 | Jackson ImmunoReseach | JI 703-545-155 |
| **Polyclonal** | Donkey | Anti-rabbit Alexa Fluor 594 | 1:200 | Jackson ImmunoReseach | JI 711-585-152 |
| **Polyclonal** | Donkey | Anti-goat Alexa Fluor Plus 488 | 1:200 | Invitrogen | A32814 |


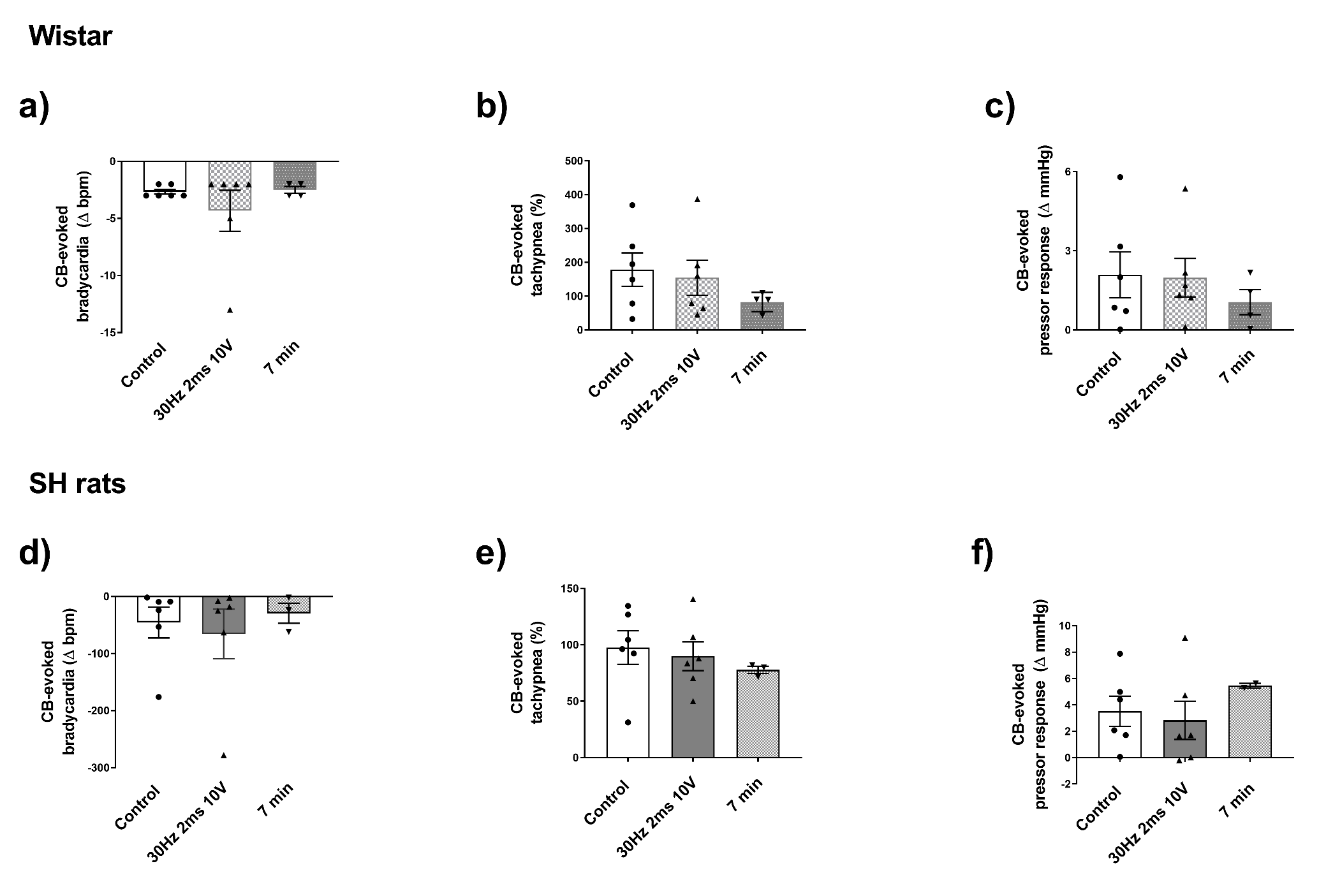


**Figure S4:** Lack of effect of ES of the SCG on the carotid body (CB)-evoked bradycardia, (a and d), tachypnoea (b and e), and hypertension (c and f) in Wistar (top graphs, n=6) and spontaneous hypertensive (SH) rats (bottom graphs, n=6). Chemoreflex was activated with sodium cyanide (NaCN 0.4 µg/µL; 50-70 µL). Data were analysed using the paired Student t-test or Wilcoxon test. Note, the reduced number of data points in panels a-f) reflects the loss of high quality recordings in 5 preparations.


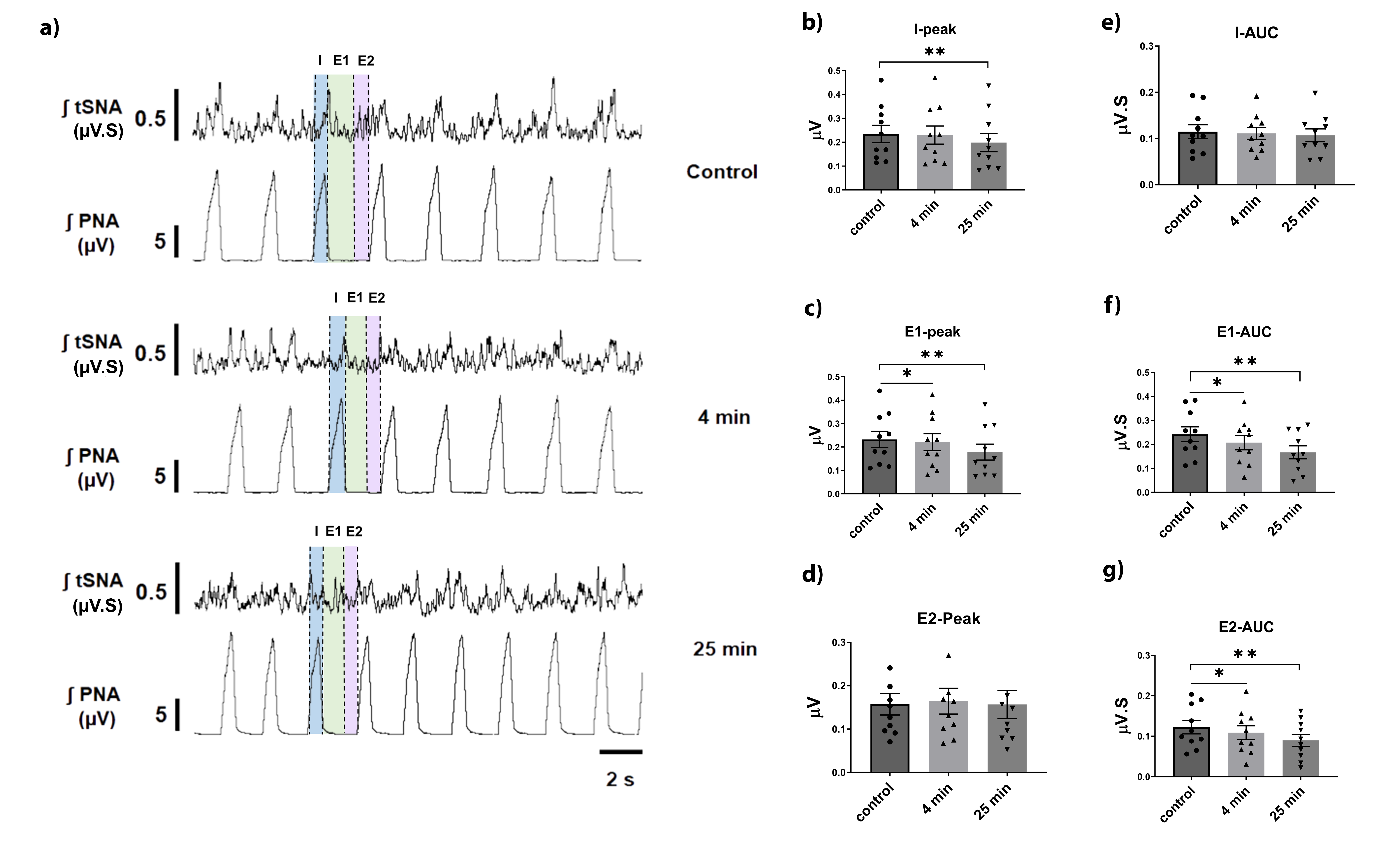


**Figure S5:** Prazosin injection (40 µL, 1 mmol/L) into the CB via the internal carotid artery reduced baseline respiratory-sympathetic coupling in spontaneous hypertensive rats (n=10). a) Integrated waveforms of phrenic (PNA) and sympathetic nerve activity (tSNA) schematically showing the different phases of the respiratory cycle. Maximum amplitude burst of sympathetic activity during the (b) inspiratory (I-Peak), (c) post-inspiratory (E1-Peak), and (d) late-expiratory phases (E2-Peak). Coupling was also measured via area under the curve (AUC) during (e) inspiratory (I-AUC), (f) post-inspiratory (E1-AUC) and (g) late expiratory phases (E2-AUC). Paired student t-test; * p<0.05 and ** p<0.01.


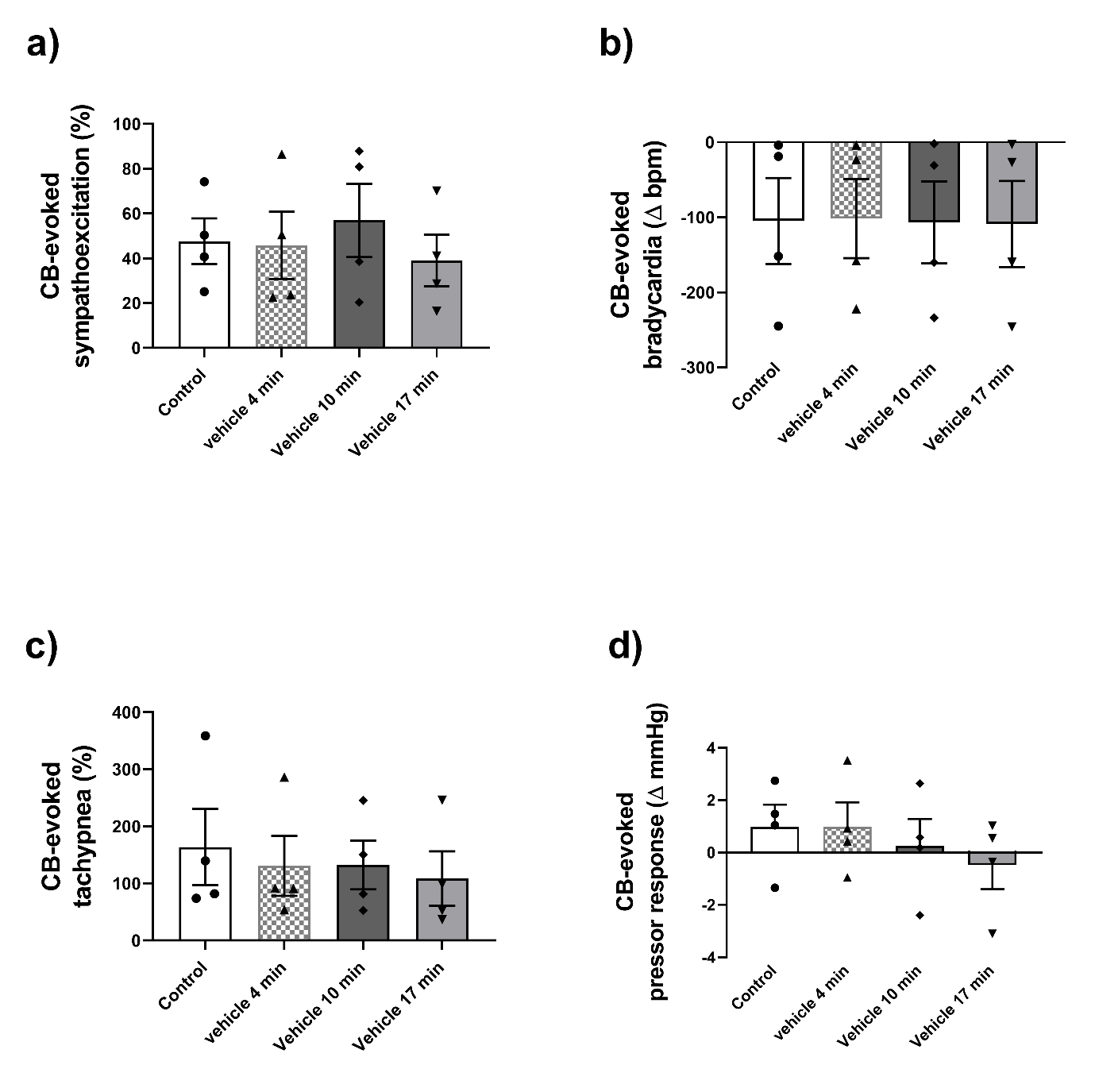


**Figure S6:** Vehicle injection (40 µL of saline pH=3) into the internal carotid artery did not change the chemoreflex response in spontaneous hypertensive rats (n=4). a) Sympathoexcitation, (b) bradycardia, (c) tachypnea, (d) pressor response. Chemoreflex was stimulated with sodium cyanide (NaCN, 0.4 µg/µL; 100 µL). Data analysed using paired Student t-test vehicle 4 min vs control and repeated measure one-way ANOVA or mixed effects model from vehicle 4 min onwards.


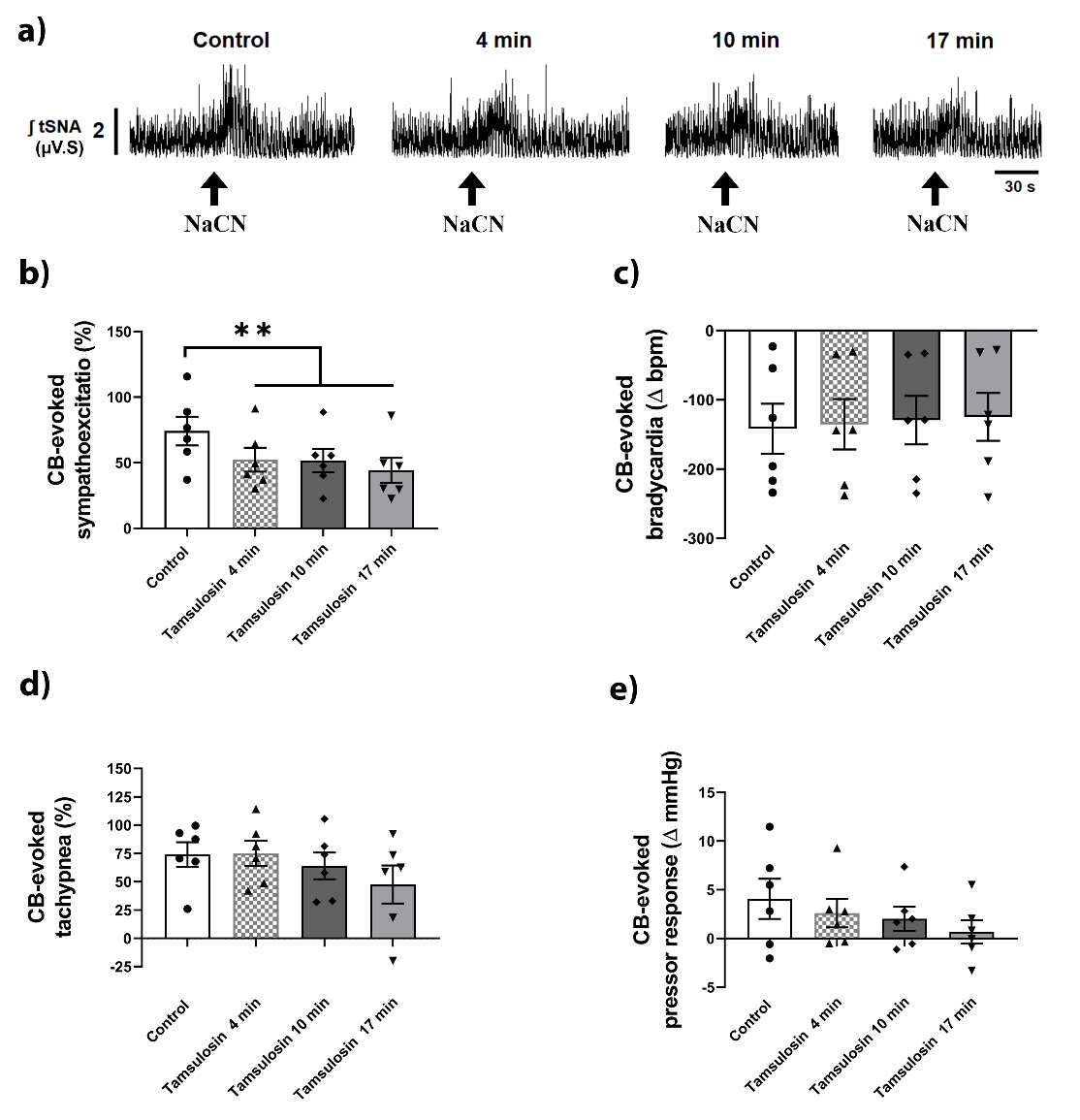


**Figure S7:** Tamsulosin injection (50 µL, 1 mmol/L) into the internal carotid artery attenuated the chemoreflex sympathoexcitation response in spontaneous hypertensive rats (n=6). a) Typical tracing of rectified and integrated thoracic sympathetic nerve activity (tSNA). Plotted carotid body (CB)-evoked (b) sympathoexcitation, (c) bradycardia, (d) tachypnea, and (e) pressor response. Chemoreflex was stimulated with sodium cyanide (NaCN, 0.4 µg/µL; 100 µL). Data analysed using paired student t-test vs control and repeated measure one-way ANOVA vs Tamsulosin 4 min; ** p<0.01 vs control.


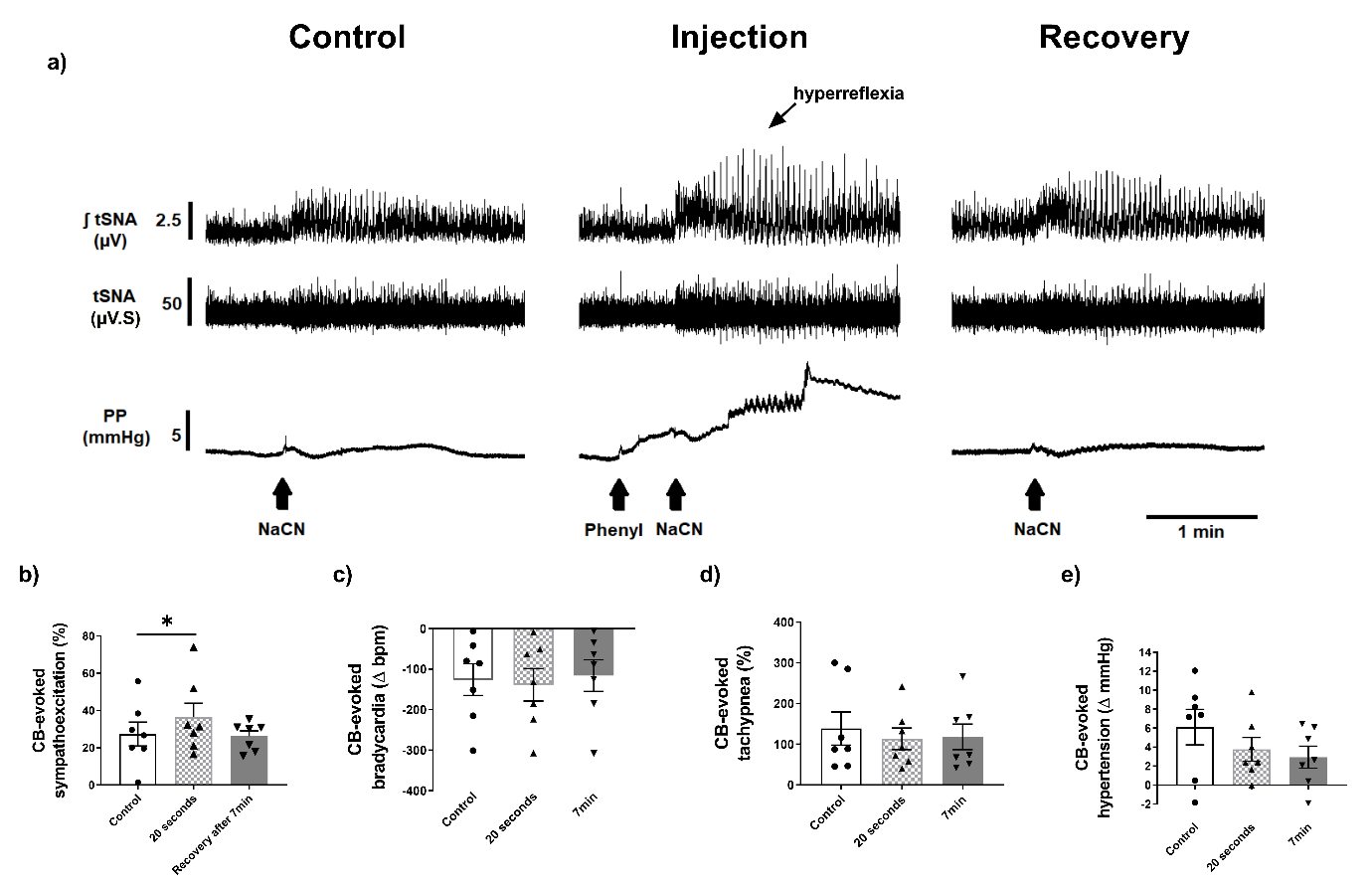


**Figure S8:** Phenylephrine injection (50 µL, 1 mmol/L) into the CB via the internal carotid artery (ICA) causes sympatho hyperreflexia in Wistar rats (n=7). Other chemoreflex evoked motor responses remained unchanged. (a) Typical tracing of the thoracic sympathetic (tSNA), and phrenic (PN) nerves (raw and integrated waveforms) alongside the changes in perfusion pressure (PP) after chemoreflex stimulation with sodium cyanide (NaCN, 0.4 µg/µL; 100 µL). Group data for chemoreflex evoked changes in sympathetic activity (b), heart rate (c), respiration (d) and perfusion pressure (e) before and after phenylephrine delivery into the CB. Data analysed using paired Student t-test or Wilcoxon test; * P<0.05 vs control.


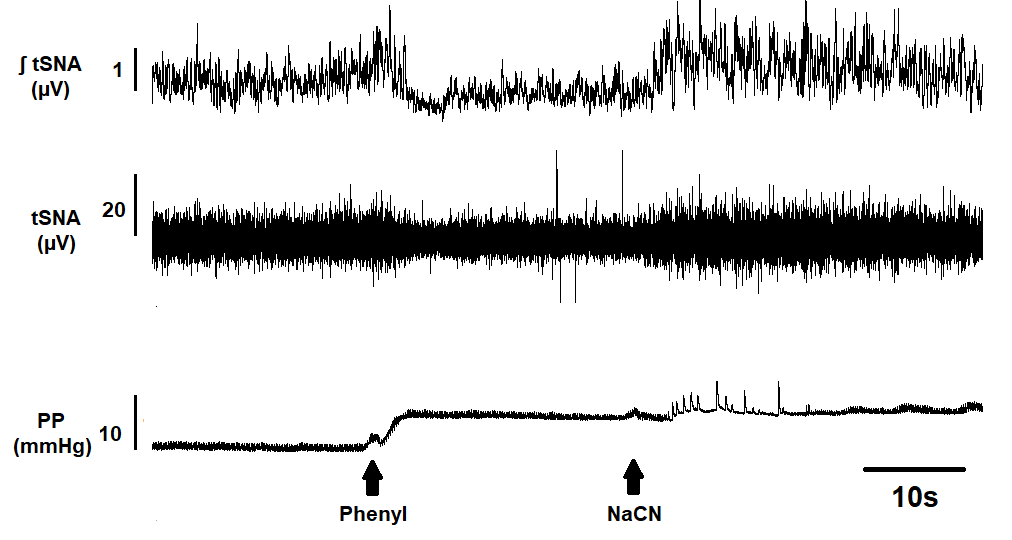


**Figure S9:** Phenylephrine injection (50 µL, 1mmol/L) into the internal carotid artery (ICA) produced baseline sympathetic inhibition baroreflex-mediated in a Wistar rat. Chemoreflex was stimulated with sodium cyanide (NaCN, 0.4 µg/µL; 100 µL).


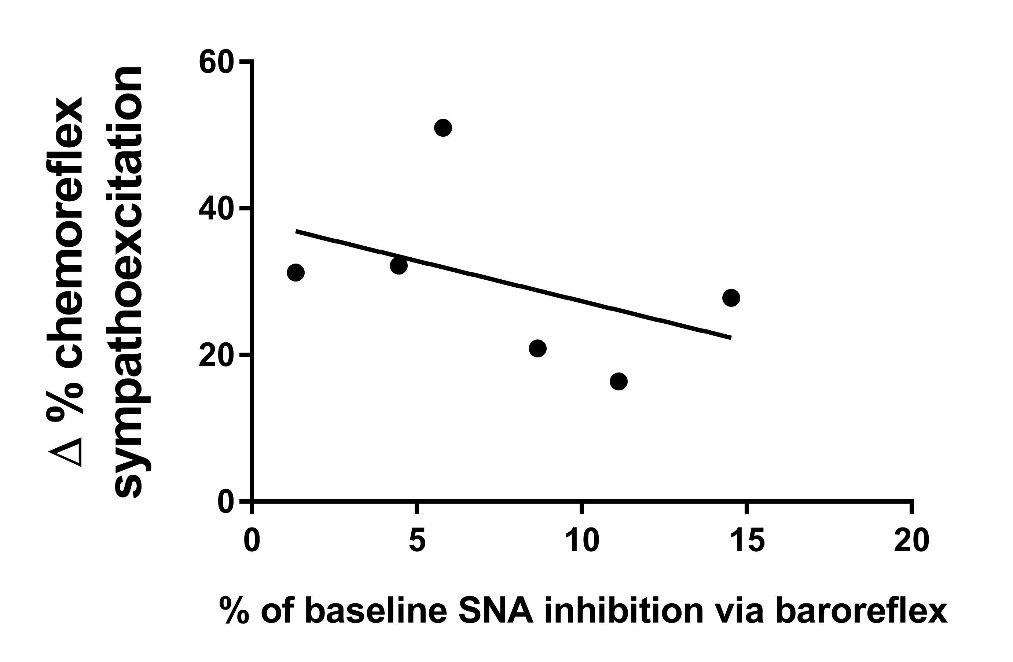


**Figure S10:** Phenylephrine sensitisation of chemoreflex sympathoexcitation seems to be negatively (r= -0.438) modulated by baroreflex activation in Wistar rats (n=6)**—**Pearson correlation analysis; P=0.384.


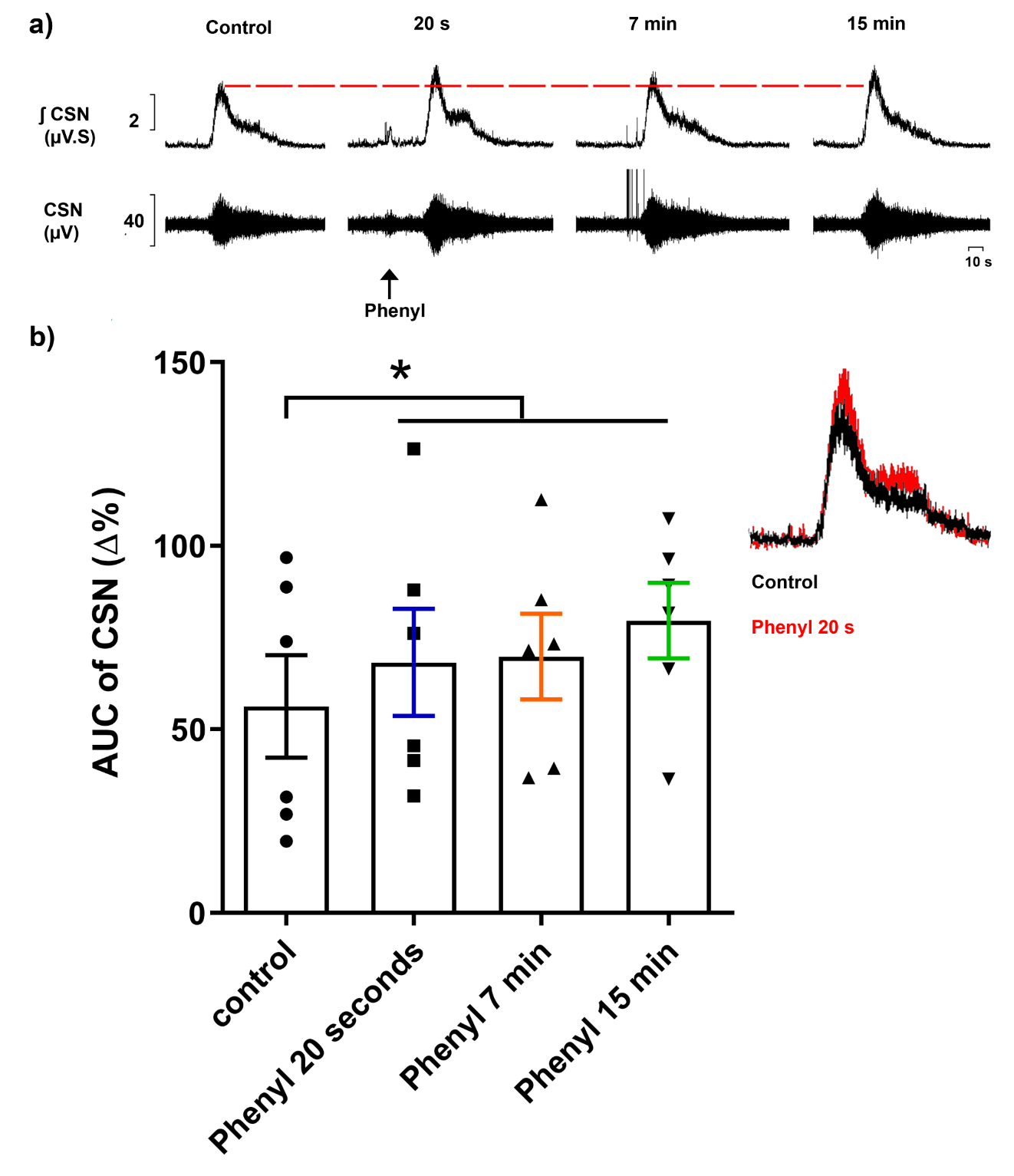


**Figure S11:** Phenylephrine injection (50 µL, 1 mmol/L) into the CB via the internal carotid artery (ICA) sensitized the CB-evoked carotid sinus nerve (CSN) discharge (n=6). (a) Typical tracing of CSN (raw and integrated waveforms) after chemoreflex stimulation with sodium cyanide (NaCN, 0.4 µg/µL 0.04%; 100 µL via aorta); on the right, superimposition of control and phenylephrine responses at 20s is shown. (b) Graph shows the percentage change in the area under the curve (AUC) after drug relative to same period of baseline. Data analysed using one-tail paired Student t-test control vs phenylephrine 20 s and RM one-way ANOVA phenyl 20 s onwards; * P<0.05 vs control.


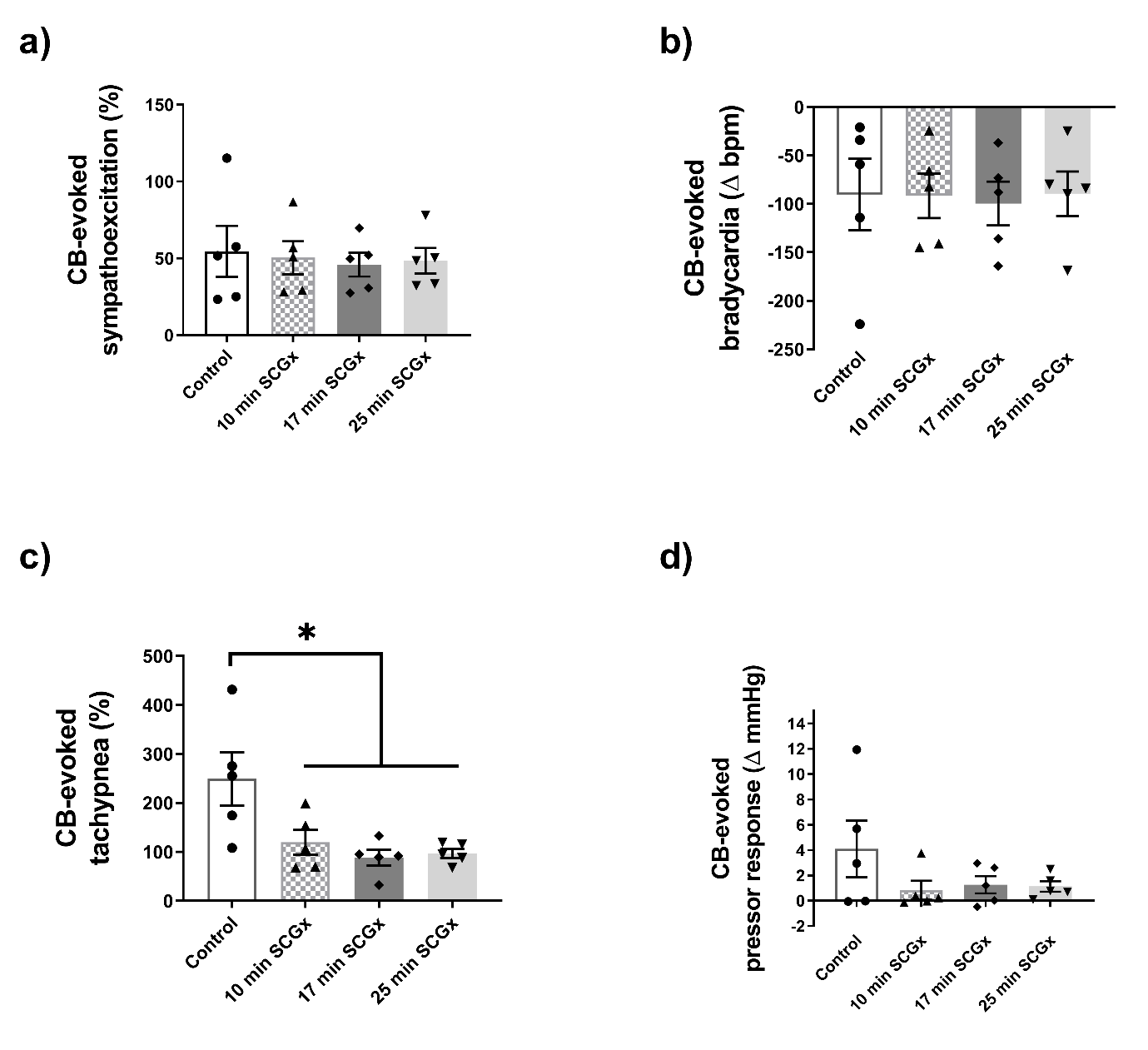


**Figure S12:** Unilateral SCG resection in Wistar rats (n=5) attenuated only the CB-evoked chemoreflex (c) respiratory response without affecting (a) sympathetic, (b) bradycardic, and (d) pressor responses. Although there is some reduction in pressor response after SCGx, this is not statistically significant. The CB-evoked chemoreflex was elicited by stimulation of the CB ipsilateral to the resection. The chemoreflex was activated with sodium cyanide (NaCN, 0.4 µg/µL; 100 µL). Data analysed using paired Student t-test or Wilcoxon test to compare control vs 10 min post-ganglionectomy and RM-ANOVA from 10 min ganglionectomy onwards; * p<0.05 vs control.


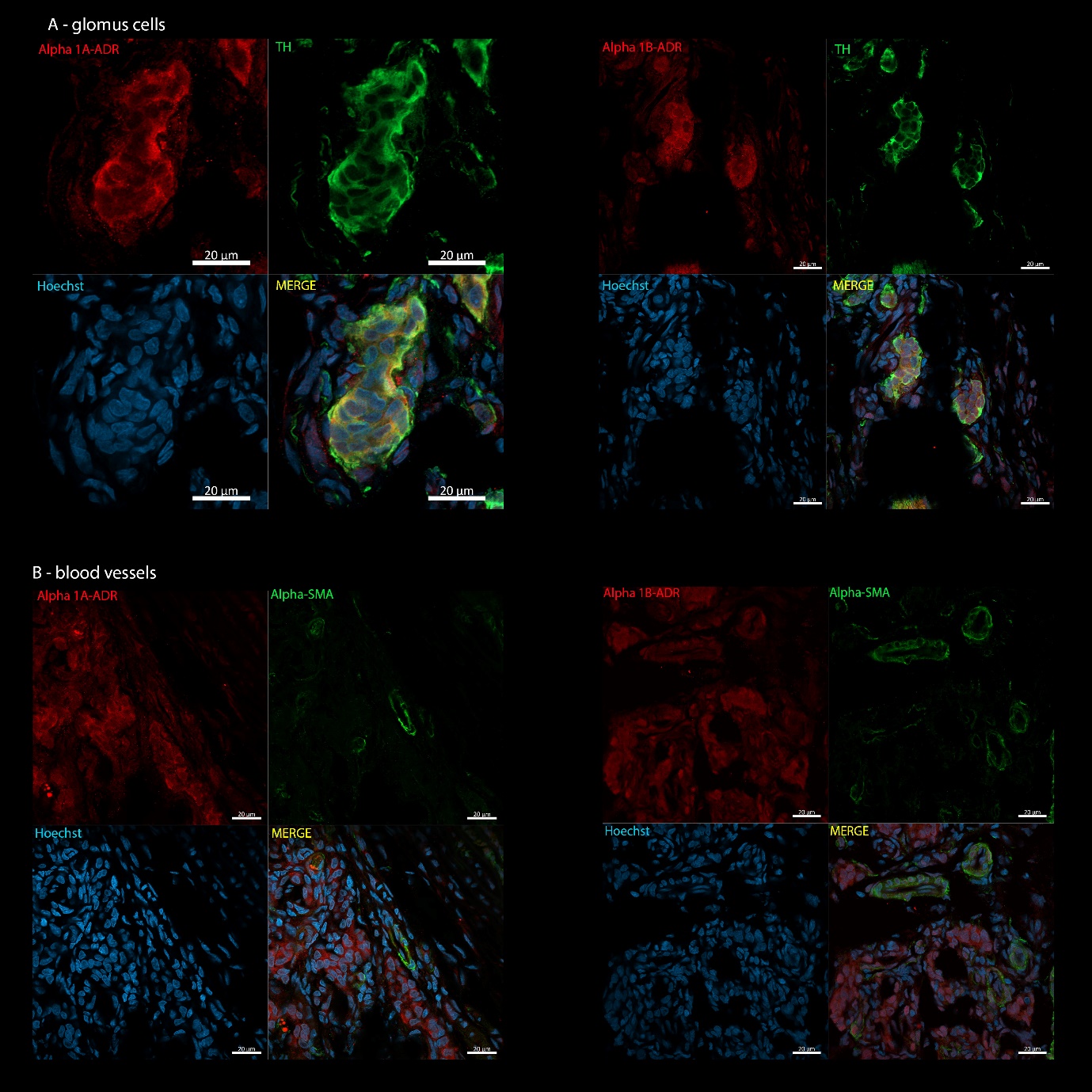


**Figure S13:** Zoom in of regions of interest displaying immunohistochemistry co-localisation in the carotid body of spontaneous hypertensive rats (SHR, n=3). A) α_1A_- and α_1B_- adrenoreceptors (Alpha 1A/B-ADR; red, Alexa Fluor 594) with tyrosine hydroxylase (TH; green, Alexa Fluor 488), a marker for carotid body chemoreceptors. B) α_1A_- and α_1B_- adrenoreceptors (Alpha 1A/B-ADR; red, Alexa Fluor 594) with α-smooth muscle actin (Alpha-SMA; green, Alexa Fluor plus 488), a marker for blood vessels. Hoechst staining is also shown for cellular nuclei (blue). Images acquired using the confocal microscope Zeiss LSM 800 Airyscan.


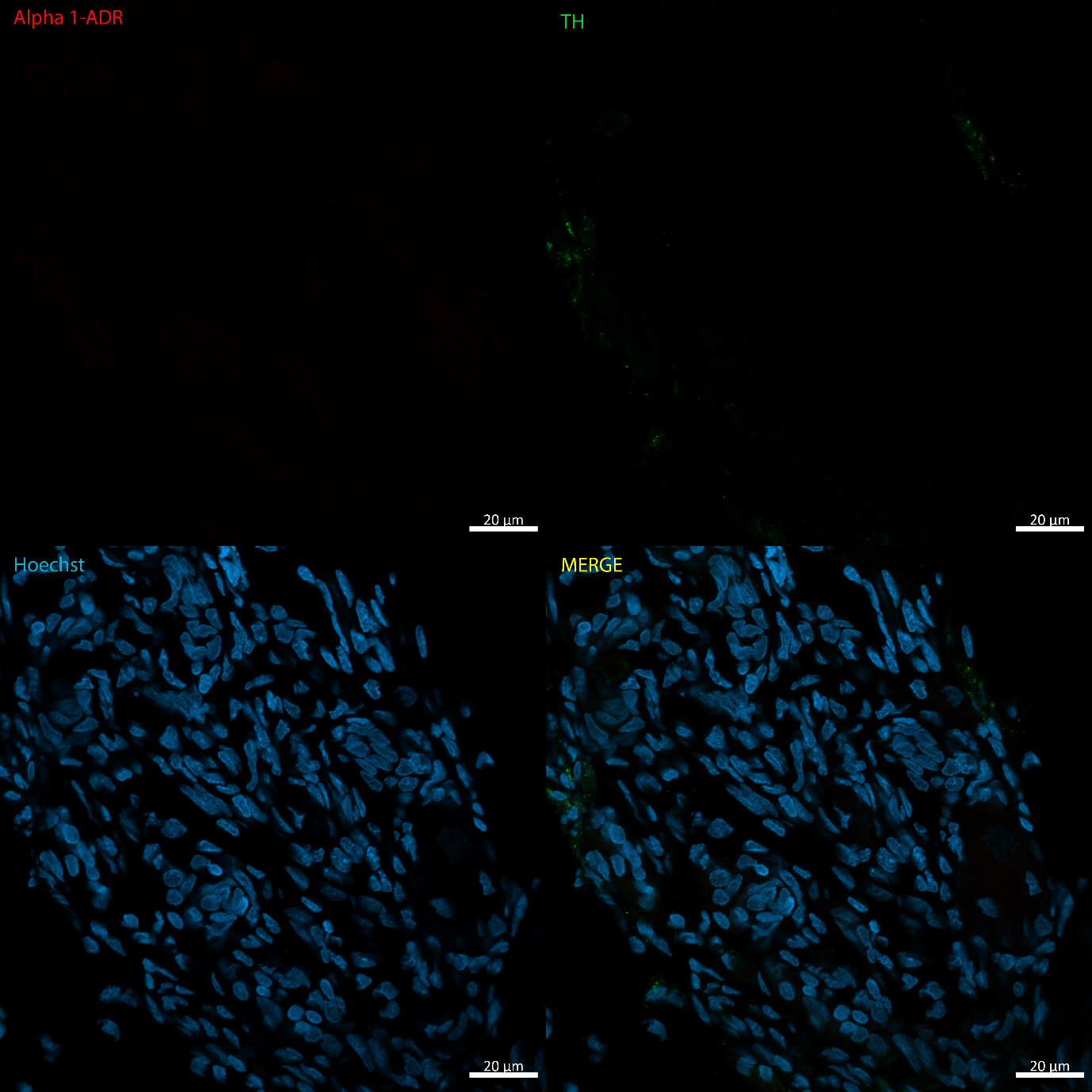


**Figure S14:** Immunohistochemistry negative control staining showing no non-specific binding for the secondary antibodies in the carotid body of Wistar rats (n=3). List of Abbreviations: Alpha 1-ADR = α_1-_adrenoreceptors (red, Donkey anti-rabbit Alexa Fluor 594), TH= tyrosine hydroxylase (green, Donkey anti-chicken Alexa Fluor 488). Hoechst staining is shown for cellular nuclei (blue) as this is incorporated in the Prolong Glass antifade media. Images acquired using the confocal microscope Zeiss LSM 800 Airyscan.


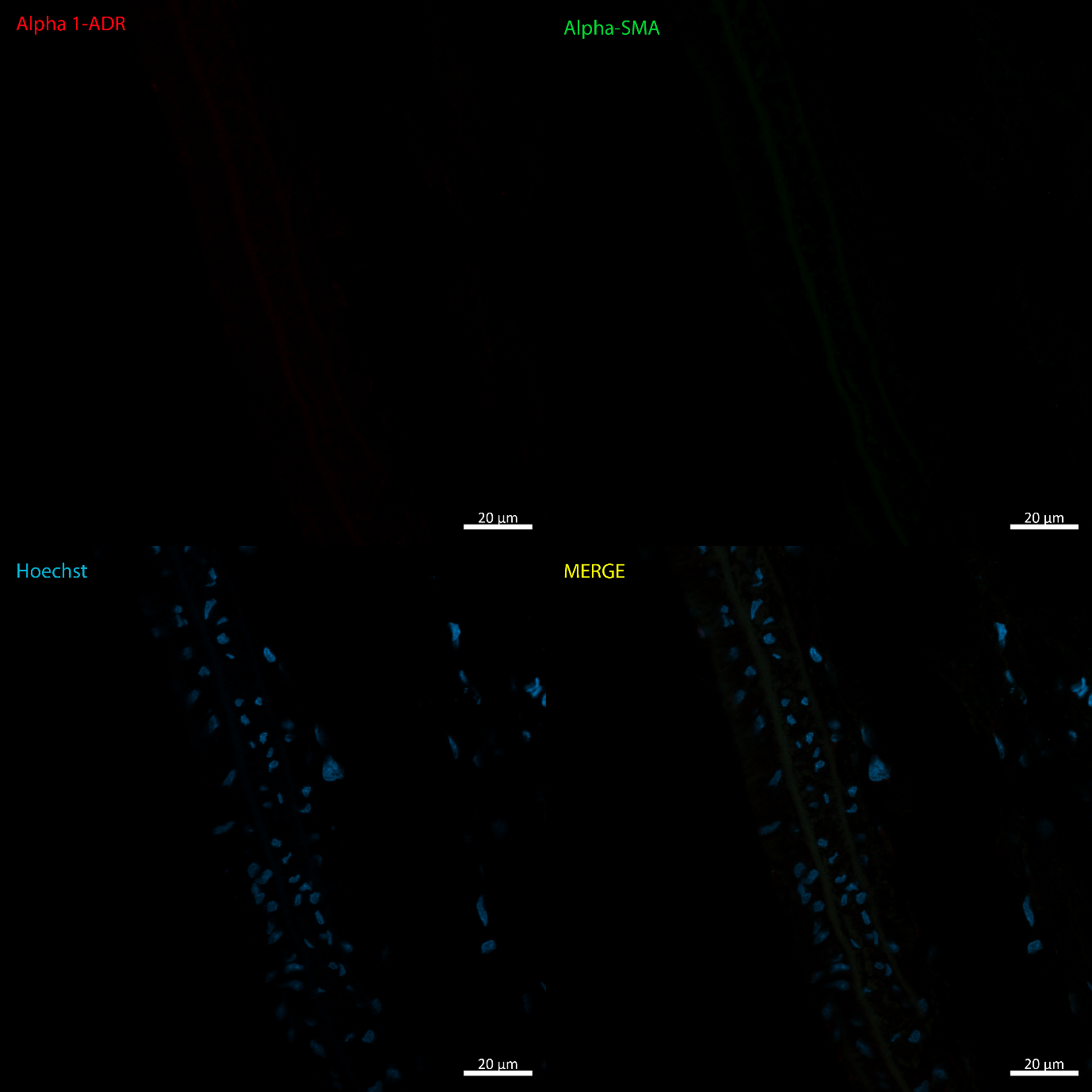


**Figure S15:** Immunohistochemistry negative control staining showing no non-specific binding for the secondary antibodies in the carotid artery bifurcation of Wistar rats (n=3). List of Abbreviations: Alpha-SMA = α-smooth muscle actin (green, donkey anti-goat Alexa Fluor Plus 488), Alpha 1-ADR = α_1-_adrenoreceptors (red, donkey anti-rabbit Alexa Fluor 594). Hoechst staining is shown for cellular nuclei (blue) as this is incorporated in the Prolong Glass antifade media. Images acquired using the confocal microscope Zeiss LSM 800 Airyscan.
